# Are European Blue Economy ambitions in conflict with European environmental visions?

**DOI:** 10.1007/s13280-023-01896-3

**Published:** 2023-08-28

**Authors:** Jesper H. Andersen, Zyad Al-Hamdani, Jacob Carstensen, Karen Edelvang, Josefine Egekvist, Berit C. Kaae, Kathrine J. Hammer, Eva Therese Harvey, Jørgen O. Leth, Will McClintock, Ciarán Murray, Anton S. Olafsson, Jeppe Olsen, Signe Sveegaard, Jakob Tougaard

**Affiliations:** 1 NIVA Denmark Water Research, Njalsgade 76, 2300 Copenhagen S, Denmark; 2 GEUS, Universitetsbyen 81, 8000 Aarhus C, Denmark; 3 Department of Ecoscience, Aarhus University, Frederiksborgvej 399, 4000 Roskilde, Denmark; 4 DTU Aqua, Kemitorvet, Building 202, 2800 Kgs. Lyngby, Denmark; 5 Department of Geosciences and Natural Resource Management, University of Copenhagen, Rolighedsvej 23, 1958 Frederiksberg C, Denmark; 6 Gladsaxe Kommune, Rådhus Alle 7, 2860 Søborg, Denmark; 7 NCEAS, 1021 Anacapa Street, Santa Barbara, CA 93101-5509 USA

**Keywords:** Cumulative pressures, Ecosystem-based management, Good Environmental Status, Marine Strategy Framework Directive, Maritime Spatial Planning, Maritime Spatial Planning Directive

## Abstract

We report the outcomes of a comprehensive study of the potential consequences of the implementation of the EU Maritime Spatial Planning Directive (MSPD) in Danish waters. The analyses are anchored in a framework developed in support of data-driven Ecosystem-Based Maritime Spatial Planning. The data for the models include not only human stressors but also information on the distribution of ecosystem components ranging from planktonic communities over benthic communities to fish, seabirds and marine mammals. We have established a baseline, based on state-of-the-art data sets, with respect to combined effects upon ecosystem components. Future scenarios for the developments in human stressors were estimated for 2030 and 2050 based on information on existing policies, strategies and plans and were compared to the baseline. In addition, we developed a scenario for implementation of the Marine Strategy Framework Directive (MSFD), i.e. working towards meeting the objectives of Good Environmental Status. Our results indicate that (1) combined human stressors will possibly increase in 2030 and 2050 compared to the baseline, (2) increased combined human stressors are likely to lead to a worsening of the environmental and ecological status sensu the Marine Strategy Framework Directive and the Water Framework Directive (WFD), and (3) the MSPD implementation process appears to conflict with the MSFD and WFD objectives. Accordingly, we are sceptical of claims of an untapped potential for Blue Growth in Danish marine waters.

**Supplementary Information:**

The online version contains supplementary material available at 10.1007/s13280-023-01896-3.

## Introduction

Maritime Spatial Planning (MSP) is the European counterpart to Marine Spatial Planning—and is a process established by the European Union’s (EU) Integrated Maritime Policy (Anon 2007), with its own legislation, i.e. the EU Maritime Spatial Planning Directive (MSPD), adopted 23 July 2014 (Anon 2014). MSP aims to reduce potential conflicts between sectors and activities competing for marine space. At the same time, MSP aims to protect the marine environment. Further, MSP intends to encourage investment by creating a level playing field between sectors and interests. MSP is defined by the UN Intergovernmental Oceanographic Commission (2021) as follows:

- MSP is a public process of analysing and allocating the spatial and temporal distribution of human activities in marine areas to achieve ecological, economic, and social objectives that have usually been specified through a political process. Characteristics of marine spatial planning include ecosystem-based, area-based, integrated, adaptive, strategic and participatory.
- MSP is not an end in itself, but a practical way to create and establish a more rational use of marine space and to manage the interactions between its uses, to balance demands for development with the need to protect the environment, and to deliver social and economic outcomes in an open and planned way.

Ecosystem-based MSP works across borders and sectors. Therefore, land-sea interactions should also be considered because human activities in upstream catchments may have significant impacts on environmental condition in downstream coastal and marine waters. For MSP implementation to be ecosystem-based, the execution process should include all ecologically relevant features and all human stressors impacting these.

Maritime Spatial Planning is in Europe considered a fundamental tool for a development of a Sustainable Blue Economy as it allows EU Member States to sustainably manage the use of coastal and marine waters to reduce conflicts, create synergies between human activities and ensure they take place in an efficient and safe way (CINEA 2023).

In Denmark, the European Maritime Spatial Planning Directive (MSPD) was adopted through the 2016 Maritime Spatial Planning Act (Anon 2016) as the basis for establishing a Danish National Marine Plan coordinating the use of marine resources and ecosystem services and sustainable Blue Growth in Danish marine waters. Towards 2030, areas will be designated for specific uses, e.g. for offshore energy production (e.g. offshore wind farms, oil and gas, CO_2_ storage), shipping, fisheries, aquaculture, sand and gravel extraction, as well as environmental protection. This upcoming national MSP plan—‘Havplan Danmark’—came into force in 2021 but is currently still in process for political approval. According to the Danish Maritime Authority (2020), ‘Havplan Danmark’ will establish not only predictable circumstances for a wide range of maritime activities but also predictable provisions regarding how sectors and their activities can use the marine space. In a Danish context, however, implementation of spatial planning at sea had to start virtually from scratch. Hence, there was an urgent need for compilation of state-of-the-art data sets, especially regarding spatial variation in stressor intensities, as well as development and testing of tools for ecosystem-based MSP.

Based the above development regarding data and tools, our study in Danish marine waters, reports on the following themes: (1) establishment a baseline for cumulative pressures upon ecosystem components in Danish marine waters by integrating state-of-the-art spatial data in to the best suitable model (i.e. EcoImpactMapper; Stock 2016), (2) estimation of the potential development in impacts for (i) 2030, (ii) 2050 and (iii) a hypothetical implementation of the EU Marine Strategy Framework Directive (MSFD; Anon 2008) in Denmark compared to the baseline, and (3) analyses of the Danish implementation of the Maritime Spatial Planning Directive with respect to cumulative impacts and their effects on environmental and ecological status. Here we use the approach developed by Halpern et al. (2008) and further refined by Halpern et al. (2009, 2015) and Stock (2016) for an additive human impact index to calculate the combined effects of human stressors as an ‘impact index’. The approach was chosen as it has, since its introduction in 2008, become a de facto global standard and now is widely used (Korpinen and Andersen 2016; Reker et al. 2020)—see section “Mapping of combined effects” for more information about the methods.

## Methods

### Study area

**Fig. 1:**
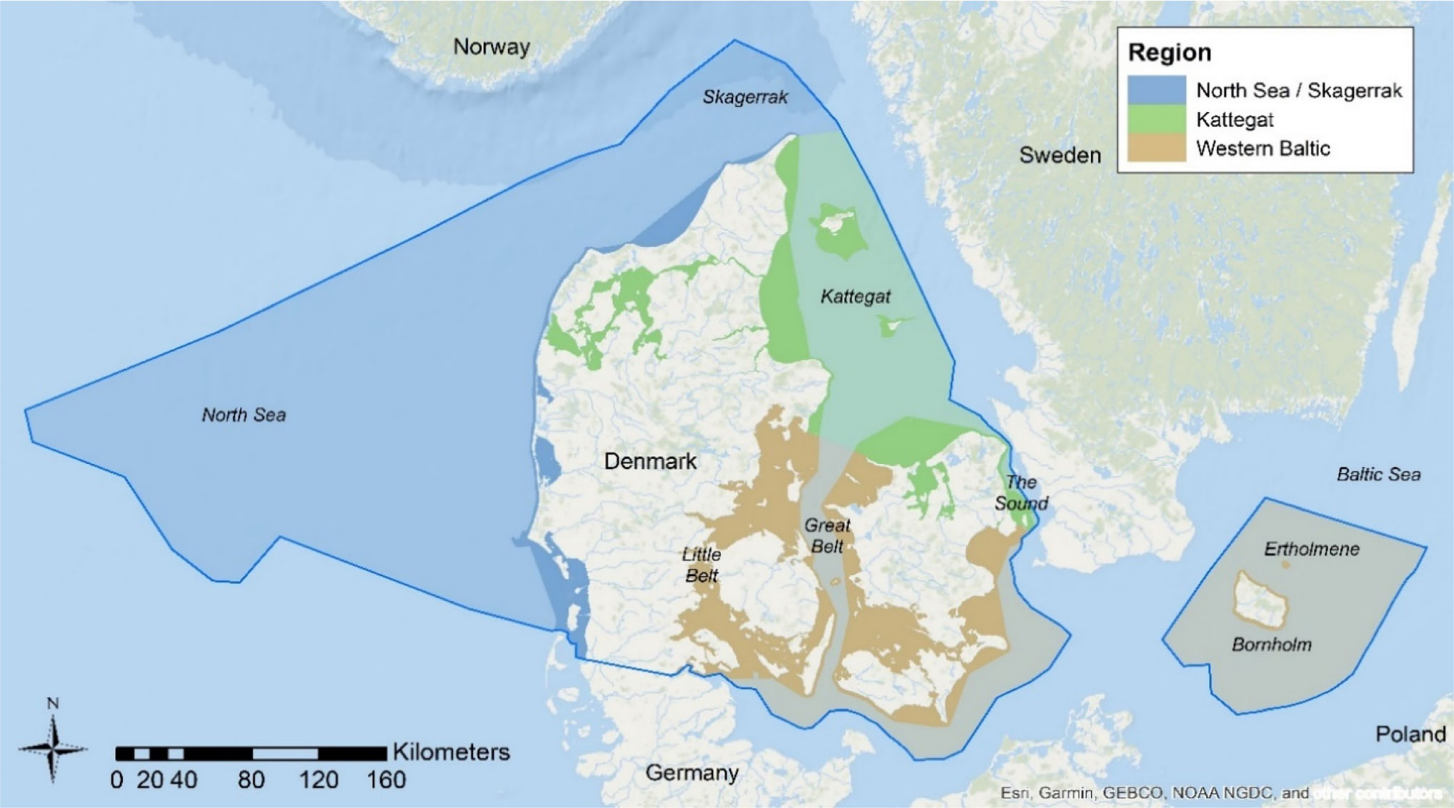
Map of the Danish Exclusive Economic Zone (EEZ). The three regions: the Danish parts of the North Sea and Skagerrak, the Danish parts of the Kattegat, and the Danish parts of the western Baltic Sea including the water around Bornholm are marked with three different colours. The darker shading indicates the area of each region within the territorial baseline + 1 nm zone (i.e. the WFD domain), the lighter shading indicates the offshore part of the regions. The Danish Marine Spatial Plan as well as the spatial data for this study cover the entire EEZ

The Exclusive Economic Zone (EEZ) of Denmark covers the eastern parts of the North Sea, the southern parts of the Skagerrak, the western and central parts of the Kattegat, the Little Belt, the Great Belt, the western parts of the Sound, parts of the western Baltic Sea and also the waters around Bornholm and the Ertholmene archipelago. The total area of the Danish EEZ is 105 000 km^2^, where coastal marine waters make up 3500 km^2^, the Territorial zone (12 nautical miles) 40 000 km^2^ and the rest of the EEZ is 61 500 km^2^. The Danish EEZ can be subdivided into three regions: the Danish parts of the North Sea and Skagerrak, the Danish parts of the Kattegat, and the Danish parts of the western Baltic Sea including the water around Bornholm (Fig. 1).

For decades, a prominent environmental problem in Danish marine waters, especially in coastal waters and the Danish Straits, has been excessive nutrient inputs and eutrophication resulting from these (Christensen et al. 1998). Nutrient enrichment has promoted high algae concentrations in surface water, reduced water transparency with associated loss of submerged aquatic vegetation, deoxygenation or even hypoxia in bottom waters, due to increasing sedimentation and mineralisation of organic matter from the surface, which occasionally have caused fish kills (see Ærtebjerg et al. 2003; Riemann et al. 2016; Uusitalo et al. 2016).

A comprehensive and cross-sectoral management plan for the Danish marine environment has been lacking. Discharges and emission of contaminants, dumping of dredged material and physical modification have been addressed separately, sector by sector (e.g. shipping, industries) or case by case (i.e. the Great Belt fixed link and the Sound fixed link). Fishing activities have been dealt with through the EU Common Fisheries Policy. Conspicuous problems associated with eutrophication, such as algal blooms, oxygen depletion and occasionally fish kills, have been overwhelming and therefore at the top of the political agenda, leaving other issues with less attention. However, with the adoption of the MSFD in 2008, the focus has widened with growing evidence that human stressors other than nutrient inputs impact Danish marine waters (Naturstyrelsen 2012; Miljø- og Fødevareministeriet 2019; Andersen et al. 2020a, b). The perspective of the human stressors in Danish marine water has evolved from a situation with a single dominant stressor (nutrient inputs) and relatively few other stressors of concern to today’s situation with a handful of dominating stressors (nutrient inputs, contaminants, commercial fishing, climate change, and physical destruction of habitats). Further, the MSFD has put focus on emerging new threats, such as introduction of non-indigenous species, inputs of marine litter and noise, thereby raising other environmental issues onto the political agenda.

Climate change, albeit an important exogenic stressor affecting marine ecosystems at large, is not directly included in the MSFD. This may seem peculiar as the MFSD is anchored in an ecosystem-based approach to management of human activities. The reason for not including climate change in MSFD is unclear, as this omission can be considered inconsistent with the definition of an ecosystem-based approach to management. However, climate change is addressed in many other ways, nationally and internationally (i.e. IPCC and EU).

### Data sources

Through cross-disciplinary cooperation, we have compiled state-of-the-art data sets for the spatial distributions of human stressors and ecosystem components (see Supplementary Information S1 and S2 for details). All relevant stressors (*n* = 42) are included along with a broad range of ecosystem components (*n* = 56) covering pelagic habitats, benthic habitats, fish, seabirds and marine mammals. These distributions constitute the basis for the application of existing tools; EcoImpactMapper (Stock 2016) and SeaSketch,^1^ but specific routines in R were also developed for post-processing of the results.

The starting point for establishing the nation-wide data set on stressors is the EU MSFD (Anon 2008), especially Annex III, Table 1 ‘Pressures and impacts’ which focuses on eight themes and 19 individual pressures. The focus in this study has been broadened (with the inclusion of data layers representing societal interests and recreation) in comparison with the MSFD and previous studies (e.g. HELCOM 2010; Korpinen et al. 2012; Andersen and Stock 2013; Riemann et al. 2016; Andersen et al. 2020a, b).

Inclusion of all ecologically relevant groups of organisms is a prerequisite for ecosystem-based MSP. Hence, this study focuses on a broad range of organisms, ranging from primary producers, i.e. phytoplankton, over a broad range of benthic habitats to top predators such as seals and harbour porpoises. The data set includes a total of seven groups and 56 individual ecosystem components.

Please see the Supplementary Information S1 and S2 for detailed information on the stressors, ecosystem components, and societal interests.

### Mapping of combined effects

Potential combined effects of human stressors as an ‘impact index’ are estimated using a concept developed by Halpern et al. (2008, 2009, 2015) and Stock (2016). The software ‘EcoImpactMapper’ (Stock 2016) is also employed in this study for the model calculations.

**Fig. 2:**
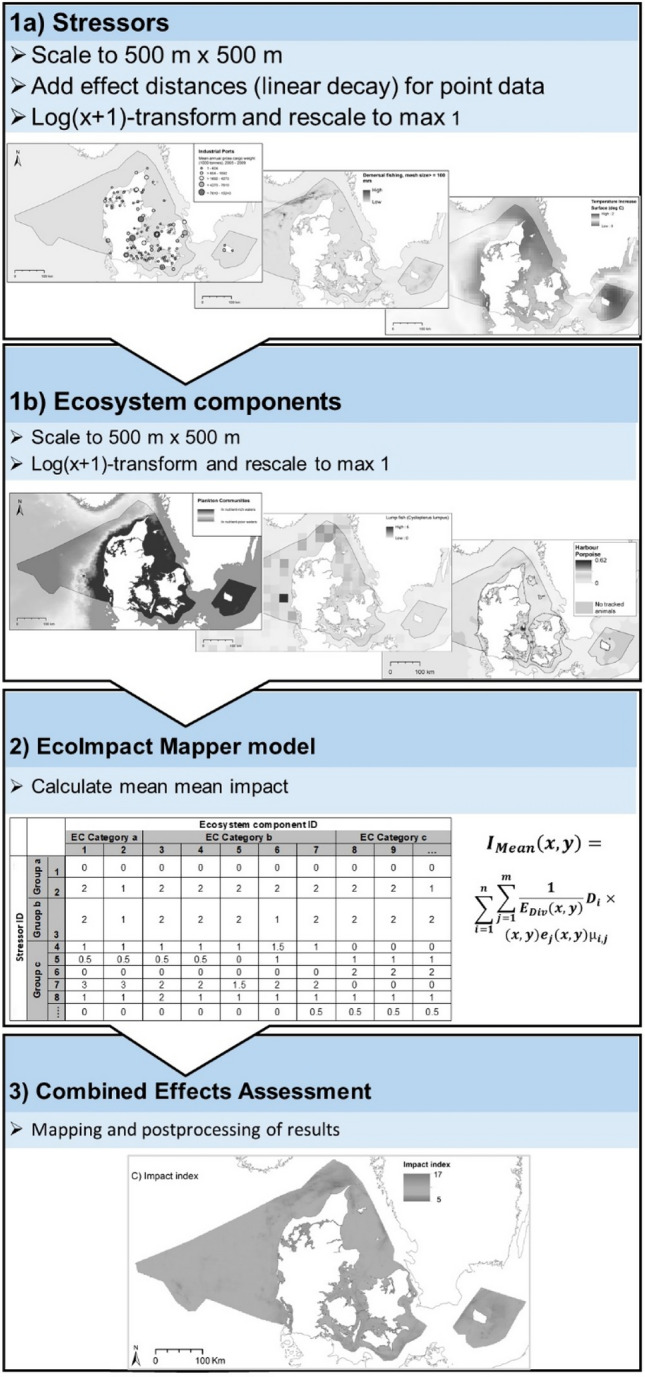
Conceptual illustration of the steps in mapping of combined effects of multiple human stressors. Step 1a: Scaling and log-transformation of individual pressure data layers including addition of effect distances for point data; Step 1b: Scaling and log-transformation of individual ecosystem component data layers; Step 2: Calculation of mean combined effects; Step 3: Mapping and subsequent post-processing of results. Data sets, effect distances and sensitivity scores are described in detail in the Supplementary Information. Based on Andersen et al. (2020a, b)

EcoImpactMapper and the Halpern approach require three kinds of input data: (1) *D*_*i*_, pressure or activity *data* (in this study merged into ‘stressors’), represented by its spatial distribution in a regular grid; for example, fishing intensity with a given gear type, (2) *e*_*j*_, ecosystem component, represented by its spatial distribution in a regular grid; for example, different broad-scale habitats, eelgrass or fish species, either as presence-absence or continuous data, and (3) *µ*_*i,j,,*_ sensitivity score, a numerical representation of the sensitivity of ecosystem component *j* to pressure or activity *i*, based on expert surveys. The intensities of the pressures and activities and the ecosystem component data were all normalized by log(*x* + 1)-transformation and rescaling to maximum 1. The reason for this was to enable comparisons between layers having different units, e.g. presence/absence, probabilities of presence, population densities or concentrations. A schematic overview of the method is presented in Fig. 2.

For stressors with a point distribution or decay from a restricted area, effect distances were also estimated based on expert surveys, and those data layers were pre-processed by adding this effect (see Supplementary Information S4). A simple linear decay function from the source and to the limit of the effect distance was used. Based on these data, we calculated the dimensionless combined effect of multiple human stressors on multiple ecosystem components for each cell in the regular grid (*x*,*y*), i.e. estimated from *n* pressures/activities, ${D}_{i}(x,y)$, and *m* ecosystem components, ${e}_{j}(x,y)$, as:

$$I_{{{\text{Mean}}}} \left( {x,y} \right) = \sum\limits_{{i = 1}}^{n} {\sum\limits_{{j = 1}}^{m} {\frac{1}{{E_{{{\text{Div}}}} \left( {x,y} \right)}}D_{i} (x,y)e_{j} (x,y)\mu _{{ij}} } }$$

where

$$E_{{{\text{Div}}}} \left( {x,y} \right) = \sum\limits_{{j = 1}}^{m} {e_{j} (x,y)}$$

In this study, the combined effects are estimated as the mean of the impact over all present ecosystem components, rather than the sum, because some ecosystem component data sets did not cover the whole study area. This mean model is also more commonly applied in more recent publications, e.g. Halpern et al. (2015) and Andersen et al. (2020a, b). The mean model is used to avoid conflating the effects of high-intensity stressors with the number of ecosystem components in each grid cell. Besides the spatially varying combined effect indices, the relative contributions of each stressor to the total effects across the study area as well as in offshore and coastal regions were also calculated.

Sensitivity scores linking specific stressors with specific ecosystem components as well as effect distances for specific point sources/stressors have been set through surveys with relevant experts. Please confer with the Supplementary Information S4 and S5 for detailed information on the sensitivity scores and effect distances.

### Scenarios

**Fig. 3:**
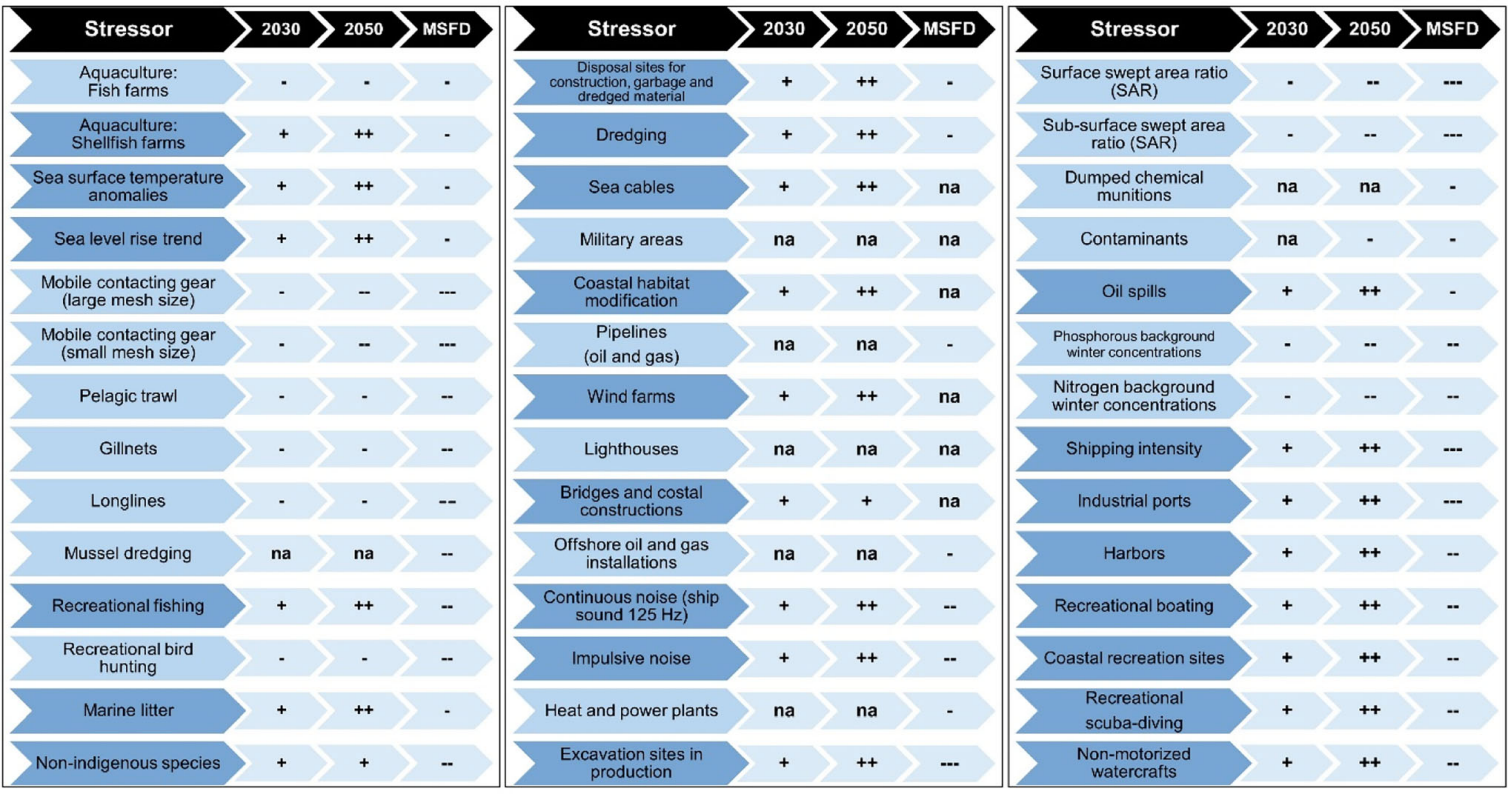
Overview of changes in stressor intensities. The signs indicate: ‘+’ = small increase, ‘++’ = moderate increase and ‘+++’ = large increase, ‘-’  = small decrease, ‘--’ = moderate decrease, and ‘---’ = large decrease. ‘na’ = not applicable/no change. Increasing pressures are highlighted with darker shading. See Supplementary Information for detailed information. Based on Andersen et al. (2020a, b)

A key objective of this study has been to analyse how changes in stressor intensities may potentially change the combined effects and subsequently improve or worsen the environmental status in Danish marine waters compared to the baseline of the status in 2018. Analyses were done for the 13 stressor groups and specific new activities were included (Fig. 3). Predicted changes were combined for two future scenarios, one for 2030 and one for 2050. Further, a scenario anchored in the ecosystem components and an improved conservation regime in accordance with the MSFD was undertaken (MSFD GES scenario). Most of the stressor changes predicted in the scenarios are directly linked to the Blue Growth strategy from the European Commission or national Danish plans and strategies, aiming to support a sustainable growth in the marine and maritime sectors. Others are projected estimates, based on the current stressors and expert inputs. In addition to the estimation of a baseline, we have combined the results and established scenarios for the years 2030 (equivalent to ‘near future’) and 2050 (equivalent to ‘long term goal’):

- 2030 scenario: We have combined what we consider is the most realistic scenario for the year 2030 and re-run the model and thus estimated how expected combined effects most likely will develop compared to the baseline.
- 2050 scenario: Similarly, as for the 2030 scenario, we have re-run the model and estimated the combined effects in year 2050 compared to the baseline, based on what we considered is the most likely scenarios for individual stressors or group of stressors. These were selected based on the identification of key pressures affecting the ecosystem component groups.
- MSFD Good Environmental Status (GES) scenario: Here the environment was prioritised by reducing the current stressor intensities from human activities with the aim of improving environmental status in accordance with the EU MSFD.

Please confer with the Supplementary Information S6 for detailed description of the changes in each pressure or activity for the 2030, 2050 and MSFD GES scenarios.

## Results and discussion

**Fig. 4:**
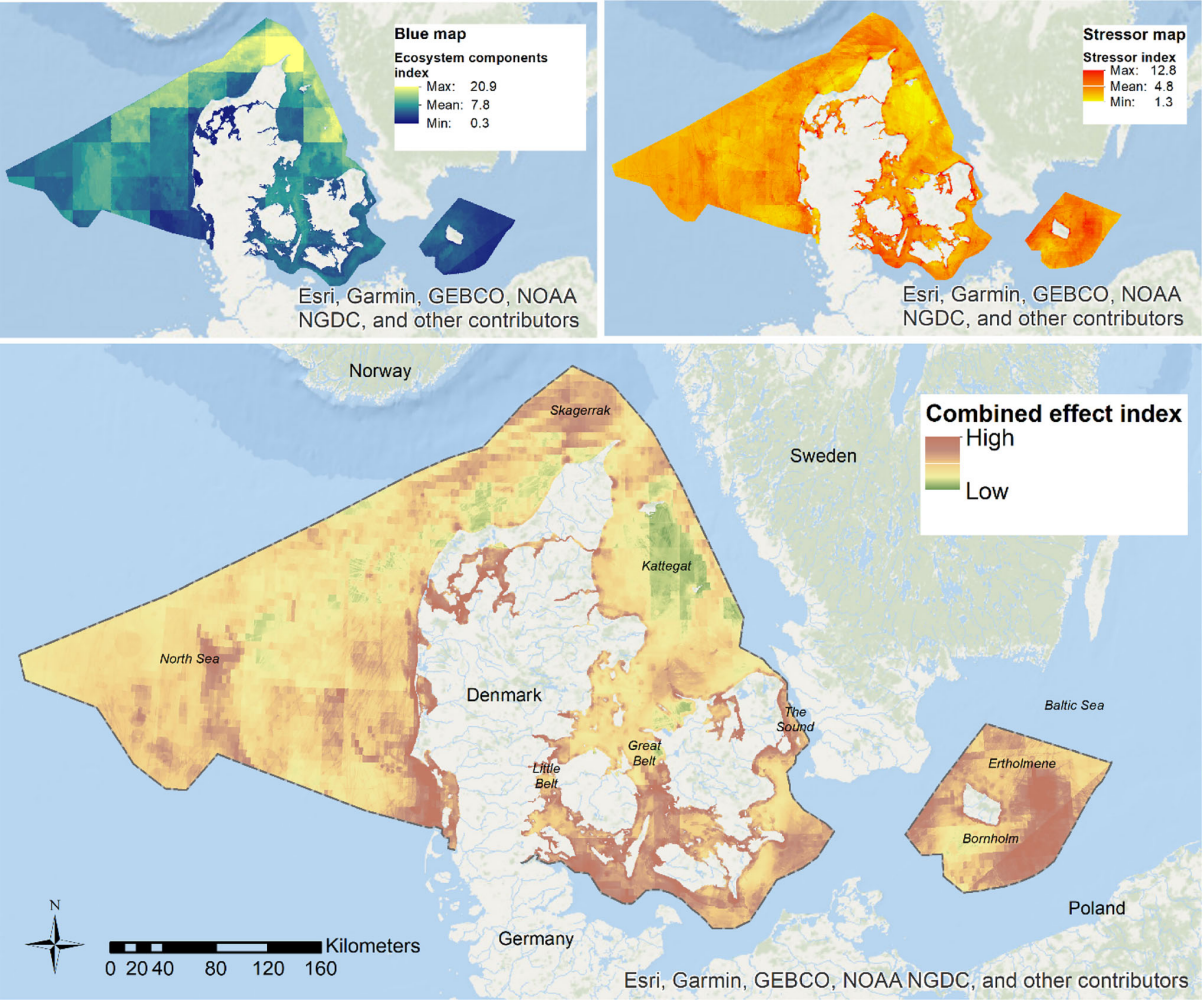
Map of intensities and spatial variations in the estimated combined effects (Combined Effect Index) based on the ecosystem components (top left panel, ‘Blue map’) and the human stressors, including climate change (top right panel, ‘Stressor map’). For method details, see Fig. 2. The colour scale shows the stretch for 2.5 standard deviations from the mean, where red indicates a higher effect impact and green lower. Note that the values are unitless and that the magnitude is defined by the model’s data inputs, which here is normalised data between 0 and 1

We report an updated nation-wide mapping of a cumulative effect assessment (CEA) (Fig. 4; see Andersen et al. (2020a, b) for details). The number of data sets included 42 for stressors and 46 for ecosystem components, a considerable expansion compared with earlier studies in Danish marine waters such as Korpinen et al. (2012), which was based on 15 stressor types and 14 ecosystem components, Andersen and Stock (2013), which was based on 33 and 28, and Andersen et al. (2020a, b), which was based on 35 and 47, respectively.

Our first step, setting a baseline, is a prerequisite for analysing the three scenarios, and for estimating differences between the scenarios and the baseline. Baselines have been produced for Danish marine waters two times, the first in 2010–2013 and the second in 2017–2018 (Naturstyrelsen 2012; Miljø- og Fødevareministeriet 2019). The baseline in this study concurs with the two previous studies, but as those were based on fewer and older data sets, the new baseline is assumed to give a better description of the spatial variation in cumulative pressures across Danish marine waters.

**Fig. 5:**
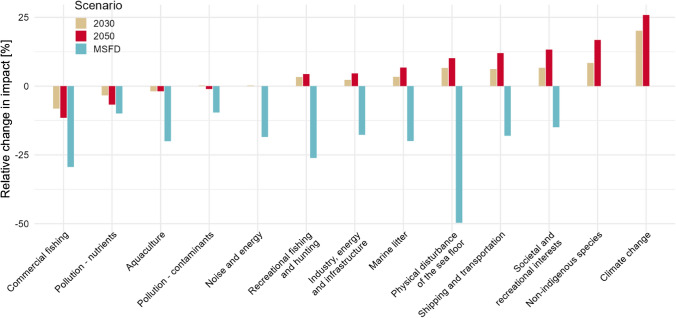
Differences between the baseline and the 2030, 2050 and MSFD GES scenarios, where 0 indicates no change, positive values an increase in the estimated combined effects per stressor group and a negative value indicates a decrease in the Cumulative Effect Assessment (CEA) per stressor group. The stressor groups are ranked according to their relative impact in the 2030 and 2050 scenarios. See Supplementary Information for a detailed description of the stressor group-specific modifications for each of the three scenarios. Please note that the changes in the scenarios are relative to the baseline impact estimated for each stressor group

Our second step, developing scenarios for 2030 and 2050 as well as a hypothetical MSFD GES scenario, should be seen as a review exercise based on existing national policies, strategies and agreed action plans in combination with implementation of both key EU directives, i.e. MSFD and WFD and implementation of regional action plans (i.e. HELCOM and OSPAR). Getting an overview of planned or expected future developments presented a substantial task. All stressors had to be analysed and supplemented with percentages representing the assumed future changes, i.e. increase, reduction or no change. The future developments are summarized per stressor group (see stressor group specific results in the Supplementary Information S7). An overview is presented in Fig. 5, where the relative changes in impacts are shown for the different stressor groups.

We have estimated changes in stressors in 2030 and 2050 to the best of our knowledge (see Supplementary Information) and not any changes in the data sets representing ecosystem components. The latter are not static, but we have chosen to rely on these as it is currently not possible to provide estimate for all ecosystem component in 2030 and 2050.

Comparing the stressor-specific effects in the 2030 and 2050 scenarios revealed diverse changes in the relative impacts at the national level, i.e. some stressor groups have reduced (e.g. commercial fishing, nutrients) while other have increased impacts (e.g. marine litter, shipping and transportation and climate change). All stressors were estimated to decrease in the MSFD scenario, except non-indigenous species and climate change (Fig. 5).

Combining stressor-specific analyses into scenarios for 2030 and 2050 and estimating the potential effects of a GES-focused reduction in stressors revealed two important findings. First, the combined effects of stressors are likely to increase in 2030 and 2050 in all waters (Table 1). Second, the MSFD GES scenario indicates a reduction in combined impacts for all types of waters and thus potentially an improvement in environmental status (Table 1). We believe this is an important result of this study. The changes revealed in the 2030 scenario yielded a total negative impact of 8%, whilst the changes revealed in the 2050 scenario yielded a total negative impact of 10.7%. In contrast, the MSFD scenario resulted in a decrease of the human impacts of 14.7%.

**Table 1:** Estimated stressor sums for all Cumulative Effect Assessment (CEA) assessment units in Danish marine waters in the baseline and in the 2030, 2050 and MSFD scenarios. *MSFD* Marine Strategy Framework Directive, *WFD* the Water Framework Directive. Please note the numbers are dimensionless

Scenario	Domain	Denmark	North Sea/Skagerrak	Kattegat	Western Baltic
Baseline	WFD coastal waters	605 263	140 793	163 474	300 997
Baseline	MSFD open waters	742 201	552 144	35 541	154 516
Baseline	Danish EEZ	1 347 465	692 937	199 015	455 512
Year 2030	WFD coastal waters	659 542	150 013	180 224	329 304
Year 2030	MSFD open waters	770 571	572 301	38 214	160 056
Year 2030	Danish EEZ	1 435 961	722 314	218 438	489 360
Year 2050	WFD coastal waters	681 160	152 449	185 687	343 024
Year 2050	MSFD open waters	779 268	577 906	39 088	162 274
Year 2050	Danish EEZ	1 466 422	730 355	224 776	505 298
MSFD GES	WFD coastal waters	514 648	123 410	139 381	251 857
MSFD GES	MSFD open waters	615 470	455 604	29 560	130 307
MSFD GES	Danish EEZ	1 130 123	579 014	168 940	382 164

When examining the spatial differences between the 2030 scenario and the CEA baseline, areas that potentially might be more affected were identified (see maps S6.1 and S6.2 in Supplementary Information). Some small, scattered areas in the central North Sea and off the Danish west coast showed small decreases of up to 10% in the potential Cumulative Effect Index. More generally, for the offshore areas there was no change or a small increase of up to 5% as the dominant change. The exceptions were in the western and southern North Sea as well as south of Bornholm, where there was an increase of 5–15% in the CEA index. Areas with a more pronounced increase in the CEA index of 15% to above 20% were found in estuaries, the Kattegat, southern Baltic Sea as well as in all the coastal areas. The large difference in the Kattegat was found in an area where the baseline CEA index was relatively low. This means that even small absolute increases in stressor levels in this area will result in proportionally high increases. More generally, according to the predictions, the largest relative increases in the CEA index in the 2030 scenario will happen in areas which have a relatively low CEA baseline index. As for the 2030 scenario, the 2050 scenario showed a general low increase with 0–5% difference for the offshore areas. However, the areas with a decrease (although low at 0–10%) were relatively larger and scattered across the North Sea and off the Danish west coast. The difference in the CEA index was larger and increasing with 10–20% or above in the same areas as in the 2030 scenario, but with wider extent and higher intensities. The largest difference was found in the southwestern Baltic Sea and the Kattegat with dominating differences increasing more than 20%. As seen in the 2030 scenario, areas with relatively low combined human stressors (low CEA index) can potentially be disturbed, so these areas could be given extra attention in the MSP plan, in addition to areas already intensively affected (high CEA index), which might not be able to absorb additional disturbances from different or new human stressors.

It follows from the above that the expectations by the EU Commission and CINEA (2023) that MSP may support the Sustainable Blue Economy and allow sustainable management of the uses of Europe’s seas to reduce conflicts and create synergies may not be fulfilled, at least for the Danish marine waters.

The MSFD GES scenario is, as mentioned, a hypothetical scenario but it does, however, demonstrate the potential effects of broadly reducing pressure intensities to improve environmental status of Danish marine waters and thus fulfil the overarching objective of the MSFD, i.e. achieving GES, or at least approach fulfilling this objective. With all effects in combination, the MSFD GES scenario resulted in a reduced impact of 14.7% overall. A general decrease in the CEA index values in the order of 10–20% is seen for the Danish EEZ but in some coastal areas in the Danish part of the North Sea, increases of more than > 20% are seen (see map S6.3 in Supplementary Information).. In the MSFD GES scenario, the only areas with increasing CEA index are designated locations for new wind farms, both under construction at present and planned in the coming years. The stressor groups ‘Non-indigenous species’ and ‘Climate change’ and their contributions to the impact intensities were assumed to be at same levels as the baseline. However, it is more realistic that they will both increase. If this happens, these pressure increases could counteract the potential positive effects of nationwide reduction in human stressors used in the scenario.

The results of the MSFD GES scenario indicate that recovery from decades of over-exploitation and pollution can be reversed and a better environmental status is within reach. Achieving this requires not only coordination but also streamlining and cross-institutional as well as cross-sector collaboration. If this is not done, a sub-optimal implementation of the MSPD could potentially lead to a reversal in some of the improvements achieved under the MSFD and WFD, as well as regional and national action plans, notably the significant progress achieved via the Danish Action Plans on the Aquatic Environment.

A recent study on the potential future combined effects of human activities including climate change is Swedish marine waters indicates that end-of-century projected climate change alone is a threat of the same magnitude as the combination of all current pressures to the marine environment (Wåhlström et al. 2022). This indicative result is probably transferable to Danish marine water and emphasize two important things: (1) Climate change may potentially be the most important pressure on marine ecosystems, and (2) if climate change is not fully included into MSP planning processes, there is a risk that there will be limited, or no, safe operating space sensu Rockstrom et al. (2009), left for introduction of new human stressors in Danish marine waters.

## Conclusions

In this study, we have demonstrated that reductions or increases in pressure intensity will have effects on the intensity of the combined stressors and ultimately on the ecosystem components. All stressor groups have been included and the potential impacts on a national level have been estimated. Based on the analyses and scenarios carried out, there is no evidence that the Danish implementation of the MSPD will actually lead to any significant improvements in ‘environmental status’ as required by the MSFD. This conclusion may be considered impetuous, but this study documents the following: (1) The combined effects in the Danish EEZ will in 2030 probably be 8% higher compared to the levels in the baseline established in this study; and (2) the combined effects will in 2050 probably be 10.7% higher compared to the levels in the baseline.

### Supplementary Information

Below is the link to the electronic supplementary material.

Supplementary file1 (PDF 1290 kb)

## Footnotes

1 www.seasketch.org.

## References

[CR1] Ærtebjerg, G., J.H. Andersen, and O.S. Hansen. 2003. *Nutrients and Eutrophication in Danish Marine Waters. A Challenge to Science and Management*. National Environmental Research Institute.

[CR2] Andersen JH, Al-Hamdani Z, Harvey ET, Kallenbach E, Murray C, Stock A (2020). Relative impacts of multiple human stressors in estuaries and coastal waters in the North Sea-Baltic Sea transition zone. Science of the Total Environment.

[CR3] Andersen, J.H., J. Bendtsen, K.J. Hammer, E.T. Harvey, S.W. Knudsen, C. Murray, J. Carstensen, I.K. Petersen, et al. 2020b. ECOMAR: A data-driven framework for ecosystem-based Maritime Spatial Planning in Danish marine waters. Results and conclusions from a development and demonstration project. NIVA Denmark report.

[CR4] Andersen, J.H., and A. Stock (eds.), S. Heinänen, M. Mannerla, and M. Vinther. 2013. *Human uses, pressures and impacts in the eastern North Sea*. Aarhus University, DCE—Danish Centre for Environment and Energy. Technical Report from DCE—Danish Centre for Environment and Energy No. 18. http://www2.dmu.dk/Pub/TR18.pdf.

[CR5] Anon. 2007. Communication from the Commission to the European Parliament, the Council, the European Economic and Social Committee and the Committee of the Regions. An Integrated Maritime Policy for the European Union. COM(2007) 575 final.

[CR6] Anon. 2008. Directive 2008/56/EC of the European Parliament and of the Council of 17 June 2008 establishing a framework for community action in the field of marine environmental policy (Marine Strategy Framework Directive). Official Journal of the European Communities L 164/19-40. https://eur-lex.europa.eu/legal-content/EN/TXT/PDF/?uri=CELEX:32008L0056&from=EN.

[CR7] Anon. 2014. Directive 2014/89/EU of the European Parliament and of the Council of 23 July 2014 establishing a framework for maritime spatial planning. Official Journal of the European Communities L 257/135-145. https://eur-lex.europa.eu/legal-content/EN/TXT/PDF/?uri=CELEX:32014L0089&from=EN.

[CR8] Anon. 2016. Lov nr 615 af 08/06/2016 om maritime fysisk planlægning. https://www.retsinformation.dk/eli/lta/2016/615 (In Danish).

[CR9] Christensen, P.B., F. Møhlenberg, L.C. Lund-Hansen, J. Borum, C. Christiansen, S.E. Larsen, M.E. Hansen, J. Andersen, et al. 1998. The Danish marine environment: Has action improved its state? - Havforskning fra Miljøstyrelsen, nr. 62

[CR10] CINEA. 2023. Maritime spatial planning in the EU. https://twitter.com/EU_MSP_Platform/status/1600488673480417281/photo/1. Accessed 08 Dec 2022 at the European MSP Platform.

[CR11] Danish Maritime Authority. 2020. https://www.soefartsstyrelsen.dk/Vaekst/Havplan. Accessed 14 Jan 2020.

[CR12] Halpern BS, Frazier M, Potapenko J, Casey KS, Koenig K, Longo C, Lowndes JS, Rockwood RC (2015). Spatial and temporal changes in cumulative human impacts on the world’s ocean. Nature Communications.

[CR14] Halpern BS, Kappel CV, Selkoe KA, Micheli F, Ebert CM, Kontgis C, Crain CM, Martone RG (2009). Mapping cumulative human impacts to California current marine ecosystems. Conservation Letters.

[CR15] Halpern BS, Walbridge S, Selkoe KA, Kappel CV, Micheli F, D’Agrosa C, Bruno JF, Casey KS (2008). A global map of human impact on marine ecosystems. Science.

[CR16] HELCOM. 2010. Ecosystem health of the Baltic Sea. HELCOM Initial Holistic Assessment 2003–2007. Baltic Sea Environmental Proceedings 122. Helsinki Commission. http://www.helcom.fi/stc/files/Publications/Proceedings/bsep122.pdf.

[CR17] IOC. 2021. https://ioc.unesco.org/our-work/marine-spatial-planning. Accessed 16 July 2021.

[CR18] Korpinen S, Andersen JH (2016). A global review of cumulative pressure and effects assessments in marine environments. Frontiers in Marine Science.

[CR19] Korpinen S, Meski L, Andersen JH, Laamanen M (2012). Human pressures and their potential impact on the Baltic Sea ecosystem. Ecological Indicators.

[CR21] Miljø- og Fødevareministeriet. 2019. Danmarks Havstrategi II. Første del. God miljøtilstilstand. Basisanalyse. Miljømål. ISBN: 978-87-93593-73-2.

[CR22] Naturstyrelsen. 2012. Danmarks Havstrategi. Basisanalyse.

[CR100] Reker, J., E.R. Gelabert, K. Abhold, S. Korpinen, C. Murray, M. Peterlin, D. Vaughan, and J.H. Andersen. 2020. Marine Messages II. Navigating the course towards clean, healthy and productive seas through implementation of an ecosystem-based approach. EEA report, 77 pp. https://www.eea.europa.eu/publications/marine-messages-2.

[CR23] Riemann B, Carstensen J, Dahl K, Fossing H, Hansen JW, Jakobsen HH, Josefson AB, Krause-Jensen D (2016). Recovery of Danish coastal ecosystems after reductions in nutrient loading: A holistic ecosystem approach. Estuaries & Coasts.

[CR24] Rockström J, Steffen W, Noone K, Persson Å, Chapin FS, Lambin EF, Lenton TM, Scheffer M (2009). A safe operating space for humanity. Nature.

[CR25] Stock A (2016). Open source software for mapping human impacts on marine ecosystems with an additive model. Journal of Open Research Software.

[CR26] Uusitalo L, Blanchet H, Andersen JH, Beauchard O, Berg T, Bianchelli S, Cantafaro A, Carstensen J (2016). Indicator-based assessment of marine biodiversity—Lessons learned from 10 case studies across the European seas. Frontiers in Marine Science.

[CR27] Wåhlström I, Hammar L, Hume D, Pålsson J, Almroth-Rosell E, Dieterich C, Arneborg L, Gröger M (2022). Projected climate change impact on a coastal sea—As significant as all current pressures combined. Global Change Biology.

