# Supplementary material for: Are European Blue Economy ambitions in conflict with European environmental visions?
Source: Ambio. 2023 Aug 28;52(12):1981–91. doi: 10.1007/s13280-023-01896-3 (PMC10654270; doi:10.1007/s13280-023-01896-3)
Supplement: Supplementary file 1 — Supplementary file1 (PDF 1290 kb) [file 13280_2023_1896_MOESM1_ESM.pdf]

Ambio

Electronic Supplementary Material
This supplementary material has not been peer reviewed.

Title: **Are European Blue Economy ambitions in conflict with European environmental visions?**

Authors: Jesper H. Andersen. Zyad Al-Hamdani, Jacob Carstensen, Karen Edelvang, Josefine Egekvist, Berit C. Kaae, Kathrine J. Hammer, Eva Therese Harvey, Jørgen O. Leth, Will McClintock, Ciarán Murray, Anton S. Olafsson, Jeppe Olsen, Signe Sveegaard, Jakob Tougaard

The Electronic Supplementary Material consists of the following:

- **S1: Pressures and human activities**
  Overview of the pressure datasets collated and applied in this study.
- **S2: Ecosystem components and societal data**
  Overview of the ecosystem component datasets as well as the datasets for societal activities collated and applied in this study.
- **S3: Effects distances**
  Table with the pressure-specific effect distances set and applied in this study.
- **S4: Sensitivity scores**
  Table with the pressures and ecosystem-specific sensitivity scores set and applied in this study.
- **S5: 2030 and 2050 scenarios and MSFD GEnS scenario**
  Overview of the changes in pressures for 13 pressure groups applied in the 2030, 2050 and MSFD GEnS scenarios.
- **S6: Results of 2030, 2050 and MSFD GEnS scenarios**
  Overview of the results from the analyses of the 2030, 2050 and MSFD GEnS scenarios for each pressure group as well as the spatial percentage difference compared to the baseline human impact assessment.
- **S7: References in Supplementary Material**

S1: Pressures and human activities

For the scenario analyses the pressures and human activities were divided into a total of 13 themes based on 42 individual pressures and activities (see detailed table below):

1. **Aquaculture:** Data on the location of marine aquaculture of fish, shellfish and plants are taken from The Danish Food Administration Agency (2020) and applied.

2. **Climate change**: Data sets on sea surface temperature anomalies and sea level increase have been adapted and applied.

3. **Industry, energy and infrastructure**: Georeferencing of the variety of human activities is based on multiple sources.

4. **Marine litter**: Data is collected under IBTS and BITS fish surveys, where several stations are trawled in a standardized procedure and where each ICES rectangle is swept representatively. In addition to the fish caught, all litter is collected.

5. **Noise and energy:** Noise is included as pulse-block days (from ICES impulsive noise register, 2016-2018) and continuous noise (ship noise levels exceeding ambient noise). Energy production is also included, following the MSFD.

6. **Non-indigenous species**: An existing index developed for MSFD reporting (Andersen et al. 2020a) has been used for ECOMAR purposes.

7. **Physical disturbance of the sea floor**: Data set on this pressure group is based on multiple sources.

8. **Pollution, contaminants**: A recent assessment of contaminants in Europe's seas (EEA 2019a) has been rescaled and used as a proxy.

9. **Pollution, nutrients**: Winter concentrations of nutrients, i.e. nitrogen and phosphorus are used as proxies for nutrient inputs and enrichment.

10. **Selective extraction of species: commercial fishing**: International landings in tons by the gear groups pelagic trawl, mobile bottom contacting gears for industrial purposes, mobile bottom contacting gears for human consumption, longlines and set gillnets as a yearly average based on the period 2015-2017. In ECOMAR, it is used as a pressure layer. VMS is mandatory or vessels larger than 12 m, so for those vessels, all fishing activity is represented in the data layers.

11. **Selective extraction of species: recreational fishing and hunting**: Data is extracted from two nation-wide surveys (Kaae, Olafsson & Draux 2018) and the activities include three types of hunting and nine types of fishing.

12. **Shipping and transportation**: Data on the spatial distribution and intensity of shipping intensities is taken from EMODnet (2020).

13. **Recreational activities**: Data come from two nation-wide surveys of 92 coastal and marine recreation activities grouped in 16 main types (Kaae, Olafsson & Draux 2018). Use frequencies are added to the approx. 16,000 mapped recreation sites and data combined with AIS data for recreational boating. Data represents the annual participation by Danes including domestic tourists, but not international tourists.

List of pressures and human activities:

# in ECOMAR report	Human pressures and activities layer	Pressure theme	Used in analyses
A1	***Pollution - Nutrients***		
A1.1	Nitrogen winter concentrations (DIN)	9	x
A1.2	Phosphorous winter concentrations (DIP)	9	x
A2	***Pollution – Contaminants***		
A2.1	Contaminants	8	x
A2.2	Dumped chemical munitions	8	x
A2.3	Oil spills	8	x
A3	***Marine Litter***	4	x
A4	***Selective extraction of species***		
A4.1	***Commercial fishing effort by gear group***	10	
A4.1.1	Set gillnet	10	x
A4.1.2	Longlines	10	x
A4.1.3	Mobile contacting gears (large mesh sizes)	10	x
A4.1.4	Mobile contacting gears (small mesh sizes)	10	x
A4.1.5	Pelagic trawl	10	x
A4.1.6	Mussel dredging	10	x
A4.2	***Recreational fishing and hunting***		
A4.2.1	Recreational fishing	11	x
A4.2.2	Bird hunting	11	x
A5	***Climate change***		
**A5.1**	Sea surface anomalies	2	x
**A5.2**	Sea level rise trend	2	x
A6	***Physical disturbance of the sea flooring***		
A6.1	***Swept area ratio (SAR) from bottom trawling***		
A6.1.1	Surface SAR	7	x
A6.1.2	Sub-surface SAR	7	x
A6.2	Extraction of material from the seafloor	7	x

# in ECOMAR report	Human pressures and activities layer	Theme	Used in analyses
A7	***Aquacultures***		
A7.1	Fish farms	1	x
A7.2	Shellfish farms	1	x
A8	***Industry, energy and infrastructure***		
A8.1	Coastal habitat modification	3	x
A8.2	Bridges and coastal constructions	3	x
A8.3	Dredging	3	x
A8.4	Disposal sites for construction, garbage and dredged material	3	x
A8.5	Offshore oil and gas installations	3	x
A8.6	Oil and gas pipelines	3	x
A8.7	Wind farms	3	x
A8.8	Sea cables	3	x
A8.9	Lighthouses	3	x
A8.10	Military areas	3	x
A9	***Shipping and transportation***		
A9.1	Shipping	12	x
A9.2	Industrial ports	12	x
A9.3	Harbours	12	x
A10	***Noise and energy***		
A10.1	Continuous noise (ship sound 125 Hz)	5	x
A10.2	Impulsive noise	5	x
A10.3	Energy production	5	x
A11	***Non-indigenous species***	6	x
A12	***Recreational activities***		
A12.1	Recreational boating	13	x
A12.2	Non-motorised watercraft	13	x
A12.3	Coastal recreation sites	13	x
A12.4	Scuba-diving recreational	13	x
			**Total 42**

S2: Ecosystem components and societal datasets

For the scenario analyses the data set of ecosystem components were divided into eight groups based on 56 individual ecosystem components (see detailed table below):

1. **Pelagic habitats**: This ecosystem component group consist of two data layers: Chlorophyll-a concentrations in surface water (used as a proxy for phytoplankton) and oxygen depletion, expressed as areas with low and very low oxygen concentrations in bottom waters. The data layer on phytoplankton is produced based on data from the joint Danish national monitoring database of the Ministry of Environment and Food of Denmark and DCE - Danish Centre for Environment and Energy, Aarhus University, for surface water data ODA (Overfladevandsdatabasen) and ICES Data Centre, the oxygen depleted data layer is based on the official reporting from the Danish centre for Environment and Energy (DCE).

2. **Benthic habitats**: This group of ecosystem components includes two types of data layers, one for brad-scale benthic habitats and one for the distribution of Eelgrass (*Zostera marina*). Information and maps on the distribution of broadscale benthic habitats originates from EMODnet and the mapping of benthic habitats in Europe's seas. In ECOMAR, we have combined the original maps in 11 groups, each being a new map. The map of the potential distribution of Eelgrass in Danish coastal waters is based on Stæhr *et al*. (2019).

The fish data set consists of three sub-groups (Sensitive fish species, Commercial fish species and Crustaceans living in benthic habitats) based on a total of 27 individual species layers. The data comes from two sources and show the distribution of fish species: one dataset shows the yearly average Catch Per Unit Effort (CPUE) per species for commercial MSFD species for the period 2015-2017. It is based on VMS data, which is only available for vessels larger than 12 m, so species caught by smaller vessels cannot be presented using this method (e.g. eel, blue mussels and cockles). Another dataset is Catch Per Unit Effort (CPUE) or Presence derived from scientific trawl surveys for the period 2009-2018, used as a proxy for abundance of commercial MSFD and sensitive red-listed species. This dataset shows the CPUE (number caught per trawl haul, standardized with respect to haul duration, year, time of the year and gear used) or Presence (probability of catching at least one individual in a standardized trawl haul). Spatial abundance indices are derived from analysis of the data from the International scientific trawl surveys, IBTS, BITS, BTS available from ICES and data from the Danish Cod and Sole surveys.

3. **Sensitive fish species:** Represent presence and absence of 12 fish species listed to be monitored under D1 biodiversity in the MSFD. Data source is national and international trawl surveys coordinated by the International Council for the Exploration of the Sea (ICES).

4. **Commercial fish species**: Represents the species distribution for MSFD 12 commercial species. Data source is national and international trawl surveys coordinated by the International Council for the Exploration of the Sea (ICES).

5. **Crustaceans:** Represents 3 commercial crustacean species under the MSFD. Data source is national and international trawl surveys coordinated by the International Council for the Exploration of the Sea (ICES).

6. **Sea birds species**: The following bird abundance data layers are included in our work: Razorbill/Guillemot (Alk/Lomvie: *Alca torda*/*Uria aalge*), Red-throated Diver/Black-throated Diver (Rødstrubet Lom/Sortstrubet Lom*: Gavia stellate*/*Gavia arctica*), Common Eider (Edderfugl: *Somateria mollissima*), Long-tailed duck (Havlit: *Clangula hyemalis*), Red-breasted Merganser (Toppet skallesluger: *Mergus serrator*), and Common Scoter (Sortand: *Melanitta nigra*). The data was

collected as part of the Danish monitoring program NOVANA. Surveys were conducted from aerial surveys by the line transect sampling method.

7. **Marine mammals**: There are data layers for three species: harbour porpoise (Marsvin: *Phocoena phocoena*), grey seal (Gråsæl: *Halichoerus grypus*), and harbour seal (Spættet sæl: *Phoca vitulina*) in the western part of the study area. However, in the North Sea and Skagerrak spatial distribution layers only exists for harbour porpoises.

8. **Recreational and archaeological interests:** The group consists of 4 individual data layers, bathing sites from EMODnet human activities, areas of recreational interests from 3 different sources, see Andersen et al. (2020b) for details and Archaeological sites, findings and Shipwrecks (archaeological and modern time) from the Danish Culture Agency.

List of ecosystem components and societal data layers:

# in ECOMAR report	Ecosystem component layer	Group	Used in analyses
B1	***Pelagic habitats***		
B1.1	Productive surface waters - chlorophyll a	1	x
B1.2	Oxygen depletion	1	x
B2	***Benthic habitats***		
B2.1*	Broad scale benthic habitats	2	x
B2.2	Eelgrass potential distribution, *Zostera marina*	2	x
B2.3	Stone reefs within \`Natura 2000´ areas	2	x
B3	***Sensitive fish species***		
B3.1	***Cartilaginous fish species***		
B3.1.1	School shark, *Galeorhinus galeus*	3	x
B3.1.2	Skates, *Dipturus spp.*	3	x
B3.1.3	Smooth-hound sharks, *Mustelus spp.*	3	x
B3.1.4	Spotted ray, *Raja montagui*	3	x
B3.1.5	Starry ray, *Amblyraja radiata*	3	x
B3.1.6	Thornback ray, *Raja claviata*	3	x
B3.2	***Bony fish species***		
B3.2.1	Atlantic wolffish, *Anarchichas lupus*	3	x
B3.2.2	Atlantic halibut, *Hippoglossus hippoglossus*	3	x
B3.2.3	Greater forkbeard, *Phycis blennoides*	3	x
B3.2.4	Ling, *Molva molva*	3	x
B3.2.5	Monkfish, *Lophius piscatorius*	3	x
B3.2.6	Rabbit fish, *Chimaera monstrosa*	3	x
B4	***Commercial fish species***		
B4.1	***Pelagic fish species***		
B4.1.1	Herring, *Clupea harengus*	4	x
B4.1.2	Mackerel, *Scomber scombrus*	4	x
B4.1.3	Norway pout, *Trisopterus esmarki*	4	x
B4.1.4	Saithe, *Pollachius virens*	4	x
B4.1.5	Sprat, *Sprattus sprattus*	4	x

# in ECOMAR report	Ecosystem component layer	Group	Used in analyses
B4.2	***Demersal/benthic fish species***		
B4.2.1	Plaice*, Pleuronectes platessa*	4	x
B4.2.2	Sole*, Solea solea*	4	x
B4.2.3	Cod,*Gadus morhua*	4	x
B4.2.4	Haddock*, Melanogrammus aeglefinus*	4	x
B4.2.5	Hake*, Merluccius merlucccius*	4	x
B4.2.6	Sandeel*, Ammodytes spp.*	4	x
B4.2.7	Turbot*, Psetta maxima*	4	x
B4.3	***Crustaceans living in benthic habitats***		
B4.3.1	Shrimp, *Crangon crangon*	5	x
B4.3.2	Norwegian lobster, *Nephrops norvegicus*	5	x
B4.3.3	Pandalus, *Pandalus borealis*	5	x
B5	***Sea birds species***		
B5.1	Auks, *Alcidae* (Razorbill/Guillemot)	6	x
B5.2	Common scoter, *Melanitta nigra*	6	x
B5.3	Eider, *Somateria mollissima*	6	x
B5.4	Fulmar, *Fulmar spp.*	6	x
B5.5	Red-breasted Merganser, *Mergus serrator*	6	x
B5.6	Red-throated/Black-throated diver, *Gavia spp.*	6	x
B5.7	Long-tailed duck, *Clangula hyemalis*	6	x
B6	***Marine mammals***		
B6.1	Grey Seal, *Halichoerus grypus*	7	x
B6.2	Harbour Seal, *Phoca vitulina*	7	x
B6.3	Harbour Porpoise, *Phocoena phocoena*	7	x
**# in ECOMAR report**	**Societal data layers**		**Used in analyses**
B7	***Recreational and archaeological interests***		
B7.1	Bathing sites	**8**	x
B7.2	Areas important for recreation and tourism	**8**	x
B7.3	Archaeological sites, findings and wrecks	**8**	x
B7.4	Shipwrecks	**8**	x
* B.2.1 includes 11 individual layers			**Total 46**

S3: Effect distances

Table showing the median, mean, maximum and minimum values (in km) for the estimated effect distances used within the CEI model. Standard deviation (km) is presented and the number of replies as well (n).

Pressure	Median	Mean	Max	Min	Stdev	n
Dumped chemical munitions	5	11.6	50	0	18.1	19
Aquacultures: fish and shellfish farms	5	10	50	0	7	20
Sea cables	0	0.10	1	0	0.37	20
Offshore oil and gas installations	1	3.5	25	0	5.77	20
Oil and gas pipelines	0	0.2	1	0	0.47	20
Heat and power plants	1	3.1	10	0	3.67	14
Disposal sites for construction and dredged material	5	8.9	50	0	12.9	20
Dredging in harbours and shipping lanes	5	6.4	50	0	12.7	14
Excavation sites in production	1	5.1	50	0	10.9	20
Offshore wind turbines	1	4.3	50	0	10.0	20
Bridges and costal constructions	1	3.2	25	0	7.5	20
Coastal habitat modification (coastal protection and piers)	1	3	25	0	6.8	14
Lighthouses	0	5.4	50	0	13.2	14
Military areas	7.5	13.4	50	0	15.3	20
Marine ports: industrial	5	10.1	50	0	14.0	14
Marine ports and marinas: recreational	3	5.6	50	0	10.9	20
Mussel dredging	1	1.2	10	0	2.6	18

S4: Sensitivity scores

Pressure-specific sensitivity scores for pelagic habitats, benthic habitats, sensitive fish species, commercial fish species, sea birds, marine mammals and recreational and archaeological interests.

	Commercial fish species															Sea birds							Marine mammals			Recreational and archeological interests			
	Mackerel	Herring	Sprat	Saithe	Norway pout	Sandeel	Cod	Sole	Plaice	Hake	Turbot	Haddock	Nephrops	Pandalus	Crangon	Auks	Red-throated/ Black-throated Diver	Common scoter	Eider	Red-breasted Merganser	Fulmar	Long-tailed duck	Grey seal	Harbour seal	Harbour Porpoise	Bathing sites	Areas important for recreation and tourism	Archaeological sites	Ship wrecks
**Pollution - Nutrients**																													
Nitrogen winter concentrations	0	1	1	1	1	1	1	1	1	0	1	0	1	1	1	0	0	0	0	0	0	0	0,5	1	1	2	1,5	0,5	0,5
Phosphorous winter concentrations	0	0,5	0,5	0,5	0,5	1	0,5	0,5	0,5	0	1	0	1	1	1	0	0	0	0	0	0	0	1	1	1	2	1	0,5	0,5
**Pollution - Contaminants**																													
Dumped chemical munitions	1	1	1	1	1	1,5	1	1,5	1,5	1	1,5	1	1	1	1	1	1	1	1	1	1	1	1	1	1	2	2	1	1
Contaminants	1,5	1,5	1,5	1,5	1,5	1,5	1,5	1,5	1,5	1,5	1,5	1,5	2	2	2	1,5	1	1	1	1	1	1	2	2	2	1,5	2	0	0
Oil spills	1,5	1,5	1,5	1,5	1,5	1,5	1,5	1,5	1,5	1,5	1,5	1,5	1,5	1,5	1,5	2	2	2	2	2	2	2	2	2	1,5	2	2	2	2
**Marine litter**																													
Marine litter	1	1	1	1	1	1	1	1	1	1	1	1	1	1	1	2	2	2	2	2	2	2	1	1	1	2	2	1	1
**Selective extraction of species- Commercial fishing effort by gear group**																													
Fishing: longlines	1	1	1	1	1	1	1	1	1	1	1	1	0	0	0	1	0	0	0	0	1	0	0	0	0	0	0	0	0
Fishing: pelagic trawl	2	2	2	2	2	1	1	1	1	1	1	1	0	0	0	1	0	0	0	0	1	0	1	1	1	0	0	0	0
Fishing: set gillnets	1	1	1	1	1	1	1	1	1	1	1	1	0	0	0	1	1	1	1	1	1	1	1	1	2	0	0	0	0
Fishing: mobile contracting gears (industrial purposes)	1	1	1	1	1	2	1	1	1	1	1	1	2	1	1,5	0	0	0	0	0	0	0	1	1	1	0	0	2	2
Fishing: mobile contracting gears (human consumption, large mesh sizes)	1	1	1	1	1	1	2	2	2	2	2	2	2	1	0,5	0	0	0	0	0	0	0	1	1	1	0	0	2	2
**Selective extraction of species - Recreational fishing and hunting**																													
Fishing: recreational	1	1	1	1	0,5	0	1	1	1	1	1	1	0	0	0	0	0	0	0	0	0	0	0	0	0	0	0,5	1	1
Mussel dredging	0	0	0	0	0	0	0	0	0	0	0	0	0	0	0	0	0	0	0	0	0	0	0	0	0	1	1	2	2
Bird hunting	0	0	0	0	0	0	0	0	0	0	0	0	0	0	0	1	1	1	1	1	1	1	0	0	0	1,5	1	0	0
**Climate change**																													
Sea surface anomalities	1	1	1	1	1	1	1	1	1	1	1	1	1	1	1	0	0	0	0	0	0	0	0,5	0,5	0,5	1	1	0	0
Sea level rise trend	0	0	0	0	0	0	0	0	0	0	0	0	0	0	0	0	0	0	0	0	0	0	0	0	0	1	1	0	0
**Physical disturbance to the seafloor**																													
Surface SAR (swept area ratio)	0	0,5	0	0,5	0	2	1	2	2	1,5	2	1	2	2	2	0	0	0	0	0	0	0	0	0	0	1	1	2	2
Sub-surface SAR (swept area ratio)	0	0,5	0	0,5	0	2	1	2	2	1	2	1	2	2	2	0	0	0	0	0	0	0	0	0	0	1	1	2	2
Extraction of material from the seafloor	0	0	0	0	0	2	0	1	1	1	1	0	2	2	2	0	0	1	1	1	0	1	1	1	1	1	1	2	2
**Aquacultures**																													
Aquacultures: fishfarms	0	0	0	0	0	0	0	1	1	0	1	0	1	0	1	0	0	0	0	0	0	0	0	0	0	1	1	1	1
Aquacultures: shellfish farms	0	0	0	0	0	0	0	1	1	0	0	0	1	0	1	0	0	0	0	0	0	0	0	0	0	1	1	1	1
**Industry, energy and infrastructure**																													
Sea cables	0	0	0	0	0	0	0	0	0	0	0	0	0	0	0	0	0	0	0	0	0	0	0	0	0	0,5	0,5	2	2
Offshore oil and gas installations	0	0	0	0	0	0	0	0	0	0	0	0	0	0	0	0	0	0	0	0	0	0	0	0	0	1	1	2	2
Oil and gas pipelines	0	0	0	0	0	0	0	0	0	0	0	0	0	0	0	0	0	0	0	0	0	0	0	0	0	1	1,5	2	2
Disposal sites for construction, garbage and dredges material	1	1	1	1	1	1	1	1	1	1	1	1	1	1	1	0	0	0	0	0	0	0	0	0	0	2	1,5	2	2
Dredging	1	1	1	1	1	1	1	1	1	1	1	0,5	2	2	2	0	0	0	0	0	0	0	0	0	1	1,5	1,5	2	2
Offshore wind turbines	0	0	0	0	0	0	0	0	0	0	0	0	0	0	0	1	1	1	1	1	1	1	0,5	0,5	1	1	1	1,5	1,5
Bridges and costal constructions	0	0	0	0	0	0	0	0	0	0	0	0	0	0	0	0	0	0	0	0	0	0	0	0	0	1	1	2	2
Coastal habitat modification	0	0	0	0	0	0	0	0	0	0	0	0	0	0	0	0	0	0	0	0	0	0	0	0	0	1	1	2	2
Lighthouses	0	0	0	0	0	0	0	0	0	0	0	0	0	0	0	0	0	0	0	0	0	0	0	0	0	0	0	0	0
Military areas	0	0	0	0	0,5	0	0	0	0	0	0	0	0	0	0	1	1	1	1	1	1	1	1	1	1	2	1,5	1	1
**Shipping and transportation**																													
Shipping	0	0	0	0	0	0	0	0	0	0	0	0	0	0	0	1	1	1	1	1	1	1	1	1	1	1	1,5	1	1
Industrial ports	0	0	0	0	0	0	0	0	0	0	0	0	0	0	0	1	1	1	1	1	1	1	1	1	1	1,5	1,5	2	2
Harbours	0	0	0	0	0	0	0	0	0	0	0	0	0	0	0	1	1	1	1	1	1	1	1	1	1	1	1	1	1
**Noise and energy**																													
Continious noise (ship sound 125 Hz)	0	0	0	0	0	0	0	0	0	0	0	0	0	0	0	1	1	1	1	1	1	1	2	2	2	0	1	0	0
Impulsive noise	0,5	0,5	0,5	0,5	0,5	0,5	0,5	0,5	0,5	0,5	0,5	0,5	0	0	0	1	1	1	1	1	1	1	2	2	2	0	1	0	0
Energy production	0,5	0,5	0,5	0,5	0,5	0	0	0	0	0	0	0	0,5	0,5	0,5	0	0	0	0	0	0	0	0	0	0	1	1	1	1
**Non-indiginous species**																													
Non-indiginous species	0	0	0	0	0	0	0	0	0	0	0	0	1	0,5	0,5	0	0	0	0	0	0	0	0	0	0	0	1	0	0
**Recreation and tourism**																													
Coastal activities	0	0	0	0	0	0	0	0	0	0	0	0	0	0	0	1	1	1	1	1	1	1	1	1	1	1	1	0	0
Non-motorised water craft	0	0	0	0	0	0	0	0	0	0	0	0	0	0	0	1	1	1	1	1	1	1	1	1	1	1	1	0	0
Boating recreational	0	0	0	0	0	0	0	0	0	0	0	0	0	0	0	1	1	1	1	1	1	1	1	1	1	1	1	1	1
Scuba-diving recreational	0	0	0	0	0	0	0	0	0	0	0	0	0	0	0	0	0	0	0	0	0	0	0	0	0	0	0	1	1

S5: 2030 and 2050 scenarios and MSFD GEnS scenario

Analyses were done for the 13 pressure groups and specific new activities were included. Predicted changes were combined for two future scenarios, one for 2030 and one for 2050. In addition, a scenario anchored in the ecosystem components and an improved conservation regime in accordance with the MSFD will be undertaken (MSFD GEnS scenario). Most of the scenarios are directly linked to the Blue Growth strategy from the European Commission or National Danish plans and strategies, aiming to support a sustainable growth in the marine and maritime sectors. Others are more general, based on the current intensities of pressures. The assumed changes for each pressure group and the 2030 and 2050 scenarios are detailly described below. The description of the scenario changes for the MSFD GEnS scenario are also described separately below. From Andersen et al. (2020c).

- **Pressure group 1: Aquaculture:** Marine aquaculture for fish meat production in net cages is a small industry in Denmark. However, this industry gives rise to environmental concerns and has consequently been on the political agenda for decades, as it releases excess nutrients and organic matter to the ecosystem and thus increasing the problems with eutrophication. According to the national aquaculture strategy production is expected to increase, mostly for land-based systems. However, increases in marine shellfish and kelp farms are also expected, which does not add but instead remove nutrients from the water column. Several estuaries have been identified as suitable for potential new shellfish farms to be established: Roskilde Fjord, Gamborg Fjord, Limfjorden, Mariager Fjord, Vejle Fjord, Kolding Fjord, Åbenrå Fjord, Augustenborg Fjord and Flensborg Fjord (Altinget 2020). Marine fish farms can expect a decrease in nutrient losses due to improved technologies and environmental concerns (Miljøstyrelsen 2020). Therefore, the 2030 scenario includes a decrease for marine aquaculture by 10% relative to present levels, but an increase in the shellfish farm area by 5% placed within the estuaries mentioned. In 2050, the increase in shellfish farm areas is 10%. Kelp farms are not considered.

- **Pressure group 2: Climate change:** Sea Surface Temperature (SST) and marine heat waves are foreseen to rise in the future if CO2 emissions are not lowered significantly (IPCC 2019). Future scenarios of SST in the North Sea (Schrum et al. 2016) and the Baltic Sea (Meier 2015) suggest an increase of about 2°C by the end of century, with some variation between the various scenarios. The rate of SST anomalies is therefore assumed to be 0.2°C per decade, which will lead to anomalies of about 0.33-0.37°C in 2030 and 0.73-0.77°C in 2050. These temperature anomalies correspond to increases of 54% in the North Sea/Skagerrak, 59% in the Kattegat and 60% in the Western Baltic in 2030, relative to baseline. In 2050, the SST anomalies will have increased by 0.6°C, which corresponds to an increase of 78% increase in the North Sea/Skagerrak, 81% in the Kattegat and 82% in the Western Baltic. The global rate of sea level rise is 3.3 mm per year (EEA 2019e, IPCC 2019) which means 3.3 cm over a decade and thus 9.9 cm increase in 2050. The change in the sea level rise trend over 10 years (2030) is assumed to be at least the same as the global rate, which then gives an increase compared the current rate of 47% in the North Sea/Skagerrak, 86% in the Kattegat and 55% in the Western Baltic. In 2050, the rate is expected to be even steeper and we used the current global rate of 3.3 mm/year times 1.5, resulting in a rate of 4.65 mm/year. For 2050 the rate is thus expected to increase compared to the current rate by 64% in the North Sea/Skagerrak, 91% in the Kattegat and 70% in the Western Baltic.

- **Pressure group 3: Industry, energy and infrastructure:** The long term forecast up to 2025 is that Denmark will be a net exporter of energy until 2035 (DEA 2018). According to this report the production will be about the same in 2030 as per today (2018), due to new findings and technical advancements, except for a temporary drop in 2020/21 due to rebuilding of a specific oil field. The wind power plants pressure is expected to increase by new areas being taken into use. The wind power parks that already are approved will be assumed to be implemented by 2030. In 2050, all areas that are “in pipeline” will be implemented (DEA 2018). An increase in wind power plants will

lead to an extension of the sea cables and accordingly, dredging within the new areas will increase and disposal sites will increase by 5% each year, which also will be implemented. An increase in shipping will also lead to an increase in coastal protections and piers with 10% in 2030 and 20% in 2050 and dredging and disposal of material will both increase by 5% in 2030 and 10% in 2050.

- **Pressure group 4: Marine litter:** Despite huge media attention and expression of political will to develop European and national plastic management strategies, there is currently no evidence supporting a decrease in the amount of plastic entering Europe's seas and Danish marine waters. On the contrary, we have assumed future trends to be increasing with 10% by 2030 and 20% in 2050.

- **Pressure group 5: Noise and cooling water:** If shipping intensity increases up to 2030, the continuous noise from shipping will also increase. Noise is measured in a log10 scale (dB) and a direct calculation of the increased noise is not possible. The 20% increase in shipping is therefore estimated to give an increase by 2.3% on average for the noise index used (based on an increase of the median level of 0.8 dB). Assuming new technologies, we estimate that by 2050 the sound pollution will decrease again and be of the same magnitude as per today. The average impulsive noise of impulse-block days per year will increase, especially in the areas where new wind power plants will be constructed, thus an increase by 20% in the new areas of wind power parks will be used for both 2030 and 2050. As no changes in inputs of cooling water to coastal waters is expected, no modelling focusing on this specific input has been undertaken.

- **Pressure group 6: Non-indigenous species:** There is no evidence for any reduction in the number in new introductions of non-indigenous species to Danish marine waters (Stæhr et al. 2016). In European Seas an average of 28 new species per year were recorded between 2006-2011 (EEA 2019f). This number is hard to directly translate to the NIS index used in the model. A reasonable and likely increase of the index by 25% over a decade and 50% for 2050, will be applied.

- **Pressure group 7: Physical disturbance of the sea floor:** Due to the expected growth in the building industry and the increased need for sand for coastal protection, the production of marine resources is expected to increase in the existing resource areas as well as in new developed areas (NIRAS, 2018). The pressure intensity is in 2030 expected to increase by 20% relative to current levels. In 2050, the areas currently designated as approved, but not active, are included in the pressure layer.

- **Pressure group 8: Pollution – Contaminants:** Oils spills are mainly from shipping and oil platforms. The risk of oil spills is proportional to shipping so their intensity is assumed to increase correspondingly with 20% in 2030 and 40% in 2050. Oil spills from oil platforms are expected to remain the same (DEA 2018). Contaminant levels are estimated to remain the same in 2030 and be reduced by 5% in 2050. It should however be noted, that there is some uncertainty regarding whether the degree of contamination is increasing or decreasing. Discharges of some substances have been reduced significantly, but at the same time, new substances are being introduced at a high rate. The data layer represents many present substances which are degraded slowly in nature. They will therefore tend to persist, even given a reduction in releases to the sea. No change in the levels of dumped chemical munitions is foreseen.

- **Pressure group 9: Nutrients:** The most important pressure in Danish marine waters, especially in estuaries and coastal waters, is nutrient input from land and atmosphere. Dedicated efforts have for decades focused on reducing losses from agriculture, discharges from urban wastewater treatment plants and industries with separate discharge. Since the late 1980s, significant reductions have been obtained for both nitrogen (appr. 50%) and phosphorus (appr. 90%). However, inputs

to coastal waters have levelled out since 2001/2002 (Andersen et al. 2019) and nitrogen inputs may even have increased in the past decade. Climate change with increased rain leading to more run-off and changes in animal production in combination with less area used for agriculture might have large impacts in the future. We assume a 10% decrease in 2030, according to the current political goals, and a slightly more ambitious goal of a decrease by 20% in 2050.

- **Pressure group 10:** Selective extraction of species - commercial fishing: Assuming that more fish species will be sustainably managed in European Seas based on the maximum sustainable yield (MSY) concept, it is expected that intensity of fishing with bottom trawl for human consumption in the Baltic Sea in 2030 will be 40% lower than at present and pelagic trawl in the same area 30% lower. Bottom trawling for industrial use remains the same. In the North Sea and Kattegat, the fishing pressure by bottom trawl for human consumption and industry are decreased by 30% each and pelagic trawl by 20%. In the 2050 scenario, the pressures are reduced with an additional 10%. The reduction of the fishing quotas for cod and sprat in 2020 and with further reductions expected in the coming years the pressure from bottom trawling will also decrease; in the Baltic Sea by 30% in 2030 and 40% in 2050; in the North Sea and Kattegat, the reduction in bottom trawling pressure is analysed with a decrease of 20% in 2030 and 30% in 2050. The pressure from other fishing methods are also reduced by 10% in 2030 and 20% in 2050. Mussel dredging occurs only in a few areas and is assumed to remain unchanged in future scenarios.

- **Pressure group 11:** Selective extraction of species - recreational fishing and hunting: In the Baltic Sea, we expect a slight reduction in the fishing of cod allowed, due to the critical condition of the population. A rise in recreational fishing in general is expected, so we have projected an increase in the order of 15% in general and a smaller increase of 10% in the Baltic Sea in 2030. For 2050 we anticipate increases of 25% and 20%, respectively. Bird hunting pressure will decrease by 10% in the western Baltic Sea due to the condition of some seabirds in 2030, in 2050 it will decrease by 20% in all regions.

- **Pressure group 12:** Shipping and transportation: Overall shipping is expected to increase by 20% in 2030 and 40% in 2050, including all types of ships from container industrial to large international cruise ships. Denmark's blue maritime strategy is investing in maritime developments to facilitate, among other things, an increase in shipping capacity. The industrial ports pressure will be increased by an equivalent of 20% and 30%, coastal protections and piers with 10% and 20% and dredging and disposal of material will both increase by 5% and 10%.

- **Pressure group 13:** Recreation and tourism: Future growth with respect to all types of tourism and recreational activities is anticipated, especially in the coastal zone. For 2030 we have increased the intensity in this pressure group with 20% for all categories and for 2050, we have increased the group with 40%.

In addition to the above pressure group-specific analyses, we have combined the results and established scenarios for the years 2030 and 2050:

- **2030 scenario:** We have combined what we consider being the most realistic scenarios for the year 2030 (see individual sections above) and rerun the model and thus estimated how the expected combined effects most likely will develop compared to the baseline.

- **2050 scenario:** Similarly, we have re-run the model and estimated the combined effects in year 2050 based on what we considered to be the most likely scenario for individual pressures or group of pressures.

DRAFT

MSFD GEnS scenario

Based on the identification of key pressures affecting the ecosystem component groups, a MSFD GEnS scenario was modelled. Here the environment was prioritised by reducing the current pressure intensities from human activities with the aim of improving environmental status in accordance with the EU MSFD. The MSFD GEnS scenario was modelled without the climate change pressure group.

- **Pressure group 1: Aquaculture** 20% decrease in current farms, and a decrease in renewed permissions. No new aquaculture introduced.
- **Pressure group 2: Climate change** is not included.
- **Pressure group 3: Industry, energy and infrastructure** Wind power remain as in 2030. Some oil and gas installations can be demounted, and the areas can be restored, as well as the pipelines -> reduction 20%. Less material dumped at sea -> reduction 30%. Dredging decrease by 20% in accordance with less shipping. Military areas explosions are set to lower levels and outside the breeding periods - reduction 20%.
- **Pressure group 4: Marine litter** reduction 20%, some of the litter can be collected and removed, better information and better control of polluters.
- **Pressure group 5: Noise and cooling water** Impulsive noise is set to a lower max limit and restricted to be outside the breeding periods- reduction by 25%. Continuous noise reduced by 2.3% related to less shipping.
- **Pressure group 6: Non-indigenous species** Better control legislation and controls of ballast water can be implemented. No change as already introduced species are present.
- **Pressure group 7: Physical disturbance of the sea floor**
- **r** Sand excavation can be reduced by 50%. No new areas are changing status to active.
- **Pressure group 8: Pollution: Contaminants** decrease by 10%, oil spills reduced 5%. Areas with dumped chemical munitions can be sanitized and bombs can be removed, reduction of 5%.
- **Pressure group 9: Pollution: Nutrients** decrease by 10% via changes of agricultural practices and land use. **Pressure group 10: Selective extraction of species: Commercial fishing** Impacts on fish species and impacts on the seafloor caused by bottom trawling decreases 30%, other fishing methods 20%. Mussel dredging reduced 10%.
- **Pressure group 11: Selective extraction of species: recreational** fishing decreases by 25%, seabird hunting by 30%.
- **Pressure group 12: Shipping and transportation** Reduction in ship traffic by 20% as well as industrial ports by 15 %. Recreational harbours remain the same.
- **Pressure group 13: Recreation and tourism** decreases by 15% due to new regulations of restricted periods for human presence.

DRAFT

S6: Results for the 2030, 2050 and MSFD GEnS scenarios

In the following section, we describe the possible consequences of modifying the pressure intensities of the 13 groups of pressures that have been altered, i.e. reduced or increased according to the above considerations and descriptions. The scenario results for each pressure group upon the eight ecosystem groups are visualised in the ECOMAR report (Andersen et al., 2020c). The spatial results of the scenarios are presented in the maps below (Figure S6.1 – S6.3), taken from the ECOMAR supplementary material (Andersen et al., 2020b).

- **Pressure group 1: Aquaculture:** The ecosystem groups most likely to be affected by aquaculture farms are pelagic habitats, benthic habitats and recreational interests. Other ecosystem components potentially affected are various species of fish and birds. The estimated impacts in the 2030 and 2050 scenarios are as follows: In 2030 and 2050 and in the MSFD scenario, improved condition may be expected regarding pelagic habitats, i.e. chlorophyll a concentration and to a lesser degree for benthic habitats and recreational interests. The MSFD scenario indicates one possibility to achieve a significant decline in impacts from aquaculture. The relation between aquaculture and plankton is well known, but it is interesting to find a relation with recreational interests as well. In the MSFD scenario, improvement could be up to three to five times greater, probably even more pronounced on a regional or local scale.

- **Pressure group 2: Climate change:** Based on the results of the mapping of combined effects and ranking of stressors, the ecosystem components likely to be affected most by climate change are pelagic habitats (increased phytoplankton biomass and lower oxygen concentration in bottom waters) and benthic habitats (e.g. submerged aquatic vegetation, biogenic reefs, and the composition of benthic invertebrates). Both the 2030 and 2050 scenarios show that climate change will lead to an increase in impact on marine ecosystems in Danish waters. This may jeopardize potential improvements likely to be obtained through reductions of other pressures. Within the MSFD GES scenario, climate change is considered as an exogenic pressure and included.

- **Pressure group 3: Industry, energy and infrastructure:** The ecosystem components and cultural interests most impacted by pressures anchored in industries, energy production and infrastructure are recreational interests, birds and benthic habitats. In the 2030 scenario, an increase in impacts can be expected due to an increase in the intensity of these activities. Groups of ecosystem components and societal interests most affected are recreational interests and benthic habitats. Other ecosystem groups to be affected are fish and crustaceans, birds and pelagic habitats. In the 2050 scenario, the most impacted groups are recreational interests, benthic habitats and birds. Even in the MSFD scenario, negative effects are envisaged, mostly regarding marine mammals, benthic habitats, birds and recreational interests. Hence, the planned activities in 2030 and 2050 and in the MSFD scenario, will result in increased impacts on marine ecosystems and most likely contribute to a further deterioration, directly or indirectly, of environmental status in Danish marine waters.

- **Pressure group 4: Marine litter:** The ecosystem groups most likely to be impacted by marine litter are birds and fish, while recreational interests, marine mammals and both benthic and pelagic communities may also be impacted. For the 2030 and 2050 scenarios, the difference between the years is directly related to the expected increase in pressure intensity. However, the MSFD reveals that reduction in pressure intensity would probably reduce the effects and subsequently lead to improvements in environmental status. It should be noted that marine litter is a rather diverse group spanning several types of litter, e.g. microplastic, plastic of different sizes and ghost nets. These sub-groups may impact different ecosystem groups in different ways – macro-litter is known to be eaten by animals, e.g. birds, while microplastic may be by eaten by filter feeders or

DRAFT

deposited at the seafloor. Knowledge about the effects for the various types of marine litter is scarce for the moment and more research on this is required to not only better understand the relationships between the effects but also to estimate potential impacts.

- **Pressure group 5: Noise and cooling water:** Reduction in noise levels is mostly linked to the reduction of local impulsive noise and will lead to reductions in the impacts on marine mammals, seabirds, and recreational interests. Minor reductions in impacts can be found for fish and benthic communities. The highest increase in impacts in the 2030 scenario is found for marine mammals, while some increase is also found for birds and recreational interests. In the 2050 scenario, only minor increases from today's levels are expected. However, the MSFD scenario indicates potentially large reductions in impacts, especially for marine mammals, fish, birds and also recreational interests. These results should be seen as provisional and require more detailed analyses and studies – if proven correct, there is an untapped potential for measures, for regulation the impulsive noise, that may ultimately improve environmental conditions for higher trophic levels, in particular marine mammals and fish. Further, the relations between reduced levels of noise and recreational interests should be scrutinized at a variety of spatial scales, e.g. sub-regionally and locally, as this pressure group may have a large influence.

- **Pressure group 6: Non-indigenous species:** The introduction of non-indigenous species (NIS) to the Danish marine environment may potentially have a large influence on its structure and functioning, as well as its species, communities and populations. In some cases, NIS can become invasive thereby acting as a significant pressure on endemic species. Substantial impacts from NIS in some parts of Danish marine waters are well-known and considered an emerging risk as the rate of newly introduced species is relatively constant (Stæhr et al. 2016). The key ecosystem components expected to be impacted by NIS are benthic habitats (including crustaceans) and recreational interests. Assuming an unchanged rate of new introductions (Fig. 11B), the 2030 scenario reveals increased impacts, while the 2050 scenario, being based on improved management practises and at the same level as today, the impacts are not surprisingly matching today's estimated impacts. No changes are seen in the MSFD scenario where already introduced species are present, and the pressure was not altered.

- **Pressure group 7: Physical disturbance of the sea floor:** Physical disturbance from a broad range of human activities is widespread in Danish marine waters, e.g. from dredging and maintenance of shipping lanes in shallow waters, exploitation of natural resources such as sand and gravel or from smothering from activities such as dumping of dredged materials from harbours and shipping lanes. With a reduction in dredging of sand and gravel in 2030 compared to today activities, a significant decrease in impacts can be expected, especially for the following ecosystem components: fish and benthic habitats including crustaceans. Some reduction in impacts are likely for pelagic habitats (reduced resuspension) as well as recreational interests. The 2050 scenario indicates a slight increase in pressure intensities impacting the ecosystem components in question almost equally, except the sensitive fish species. Given that significant improvements are attained in a short-term perspective and not in a long-term, both political focus and more research on the environmental consequences of dredging of sand and gravel as well as smothering is urgently required.

- **Pressure group 8: Contaminants:** Discharges and losses of contaminants from Danish sources in combination with long-range transport and deposition constitute important pressures for the Danish marine environment. Multiple strategies and action plans have been adopted and implemented, presumably with a variety of successes (Dahlöf & Andersen 2009). In the 2030 scenario, where the pressure intensity is assumed to increase slightly, the impact will increase with regard

to fish and crustaceans, but also marine mammals. The 2050 scenario may, however, lead to reductions in the pressure intensity and thus a lower impact on marine mammals, fish, benthic habitats and birds. The MSFD scenario, focusing on attaining a better environmental status through a major reduction of pressure intensity, indicates lower impacts on the following ecosystem component groups: fish, marine mammals, benthic habitats, and seabirds. The latter indicates that reductions of inputs of contaminants are essential for higher trophic levels to improve the currently impaired conditions and to meet both the objectives of the MSFD and WFD as well as the so-called Generation Target.

- **Pressure group 9: Nutrients:** Nutrient inputs resulting in elevated nutrient concentration and eutrophication effects have for decades been a crucial pressure in Danish marine waters, especially in estuaries and coastal waters. Significant efforts have been made to reduce nutrient inputs from agriculture, urban wastewater treatment plants and from industries with separate discharge (see Andersen 2012 and Riemann et al. 2016). The 2030 and MSFD scenarios are identical and the groups of ecosystem components most likely to face reduced impacts are pelagic habitats (i.e. chlorophyll a concentration in surface water and oxygen concentrations in bottom waters) and benthic habitats including the key species eelgrass. In the 2050 scenario is based in reduction in nutrient inputs and thus lower nutrient levels is surface waters, highlights that significant reduction in pressure intensity and subsequently impacts on pelagic and benthic habitats. This indicates that improvement in both coastal waters (WFD domain) and offshore water (MSFD domain) can be expected – this should support implementation of additional measures and reduction in nutrient inputs. Follow up analyses focusing on specific coastal waterbodies, specific ecosystem components groups (related to WFD biological quality elements or the MSFD D5 descriptor) are urgently needed.

- **Pressure group 10: Selective extraction of species: commercial fishing:** A growing number of studies and reports on human activities and pressures in Danish marine waters have indicated that fishing, especially bottom trawling, is a significant pressure (Miljø- og Fødevareministeriet 2019, HELCOM 2018, Andersen et al. 2020a, EEA 2020). These results are confirmed by the analyses done in the context of ECOMAR. In 2030, assuming a reduction in fishing intensity, reduction in impact may be expected for the following ecosystem groups: fish, crustaceans and benthic habitats. Some effects, but to a lesser extent, are foreseen for pelagic habitats, marine mammals (due to bycatch) and recreational interests. The 2050 scenario is parallel to the 2030 scenarion with slightly higher reduction in pressure intensity, whilst the MSFD scenario indicate that significant reduction in pressure intensity.

- **Pressure group 11: Selective extraction of species: recreational fishing and hunting:** Given the availability of information on recreational fishing, we have tentatively estimated the potential effects of changes in the intensity of this specific activity. Assuming a slight increase in recreational fishing in 2030, an increased impact is seen on fish populations, in benthic habitats and for recreational interests. In the 2050 scenario, the ecosystem components estimated to encounter reduced impacts are fish and crustaceans as well as sea birds, mammals and benthic habitats. The MSFD scenario indicates reductions in the pressures on the following ecosystem components: seabirds and fish as well as recreational interests. Although being a pressure of restricted importance on a national scale, recreation can be of significant importance locally, for example in Øresund. There is a need for more detailed studies, also linking the status and pressures of target fish species to environmental conditions.

- **Pressure group 12: Shipping and transportation:** Shipping can impact a broad range of ecosystem components through its presence, by resuspension of material at the seafloor or by generating waves etc. Accordingly, the key ecosystem component groups impacted are seabirds, marine

mammals and benthic habitats. Recreational interest can also be affected. The 2030 scenario indicates elevated levels of impacts for seabirds, recreational interests, marine mammals and benthic habitats, the latter probably through physical effects. In the 2050 scenario, the same ecosystem component groups will be even more impacted. In the MSFD scenarios, the ecosystem groups assumed to face a reduction in the impacts are the same.

- **Pressure group 13: Recreation and tourism:** Recreational activities and tourism primarily have impacts on seabirds, marine mammals, benthic habits, and other recreational interests. In the 2030 and 2050 scenarios, the pressures are assumed to increase and so are the potential impacts on the ecosystem groups and recreational interests. Reductions of recreational activities and tourism, and envisage in the MSFD scenario, will lower the impacts on seabirds, marine mammals, recreational interests and benthic habitats.

Results from the combined scenarios:

Figure S6.1 shows the percentage difference between the baseline impacts to the 2030 scenario. Within the Danish EEZ only an increase in the impact and no decrease could be seen, especially with large increase in all coastal areas with more than 20 % increase; Smålandshavet (south east), south of Bornholm, Wadden Sea (south western North Sea), eastern North Sea, within Limfjorden, Aalborg bay (east of Jylland, western Kattegat) as well as in offshore areas in western North Sea.

The results of the 2050 scenario are even more pronounced (Figure S6.2) with a substantial part of the EEZ area showing more than a 20% increase in impact. The same areas, including the coastal zone, as in the 2030 scenario show the largest increase in impact towards the current situation; Smålandshavet (south east), south of Bornholm, Wadden Sea (south western North Sea), eastern North Sea, within Limfjorden, Aalborg bay (east of Jylland, western Kattegat) and western North Sea offshore areas.

'For the ˈenvironment firstˈ MSFD GEnS scenario (i.e. where the environment is prioritized) (Figure S6.3) a decrease was seen within all of the Danish EEZ areas, where a 20% change or higher decrease is indicated by dark blue areas. A substantial part of the EEZ shows a small decrease in the change in impact, between 0-5 % (light green area). The highest improvements were seen in many of the fishing grounds. An increase in impact was only seen in the areas of new wind farms.

DRAFT

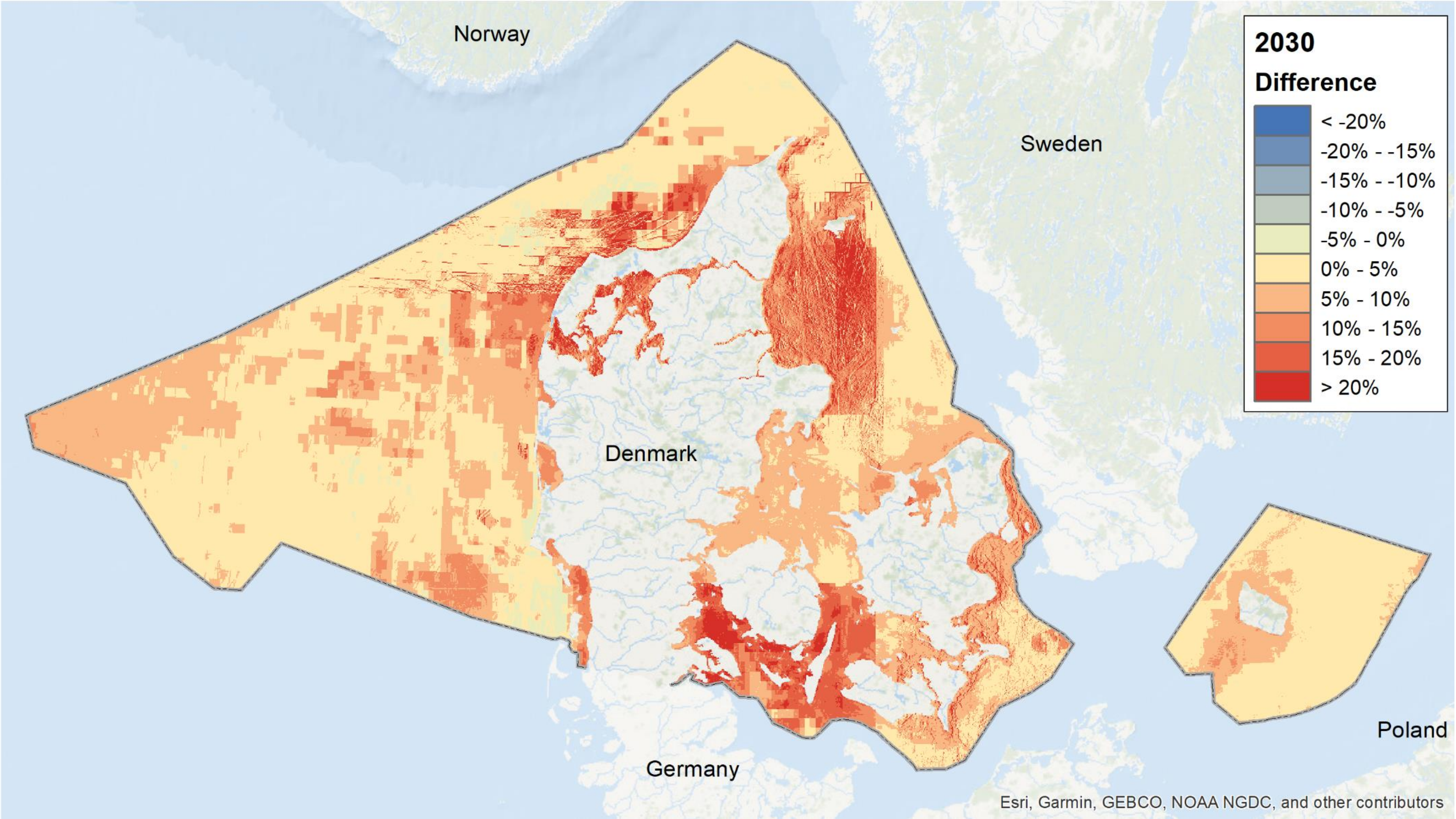

Figure S6.1. Spatial map showing the percentage difference between the baseline current impacts and when the changes in scenario 2030 are applied. See legend for colour coding, where yellow to red colours indicate an increase and green to blue a decrease in human impacts. Image from Andersen et al. (2020b).

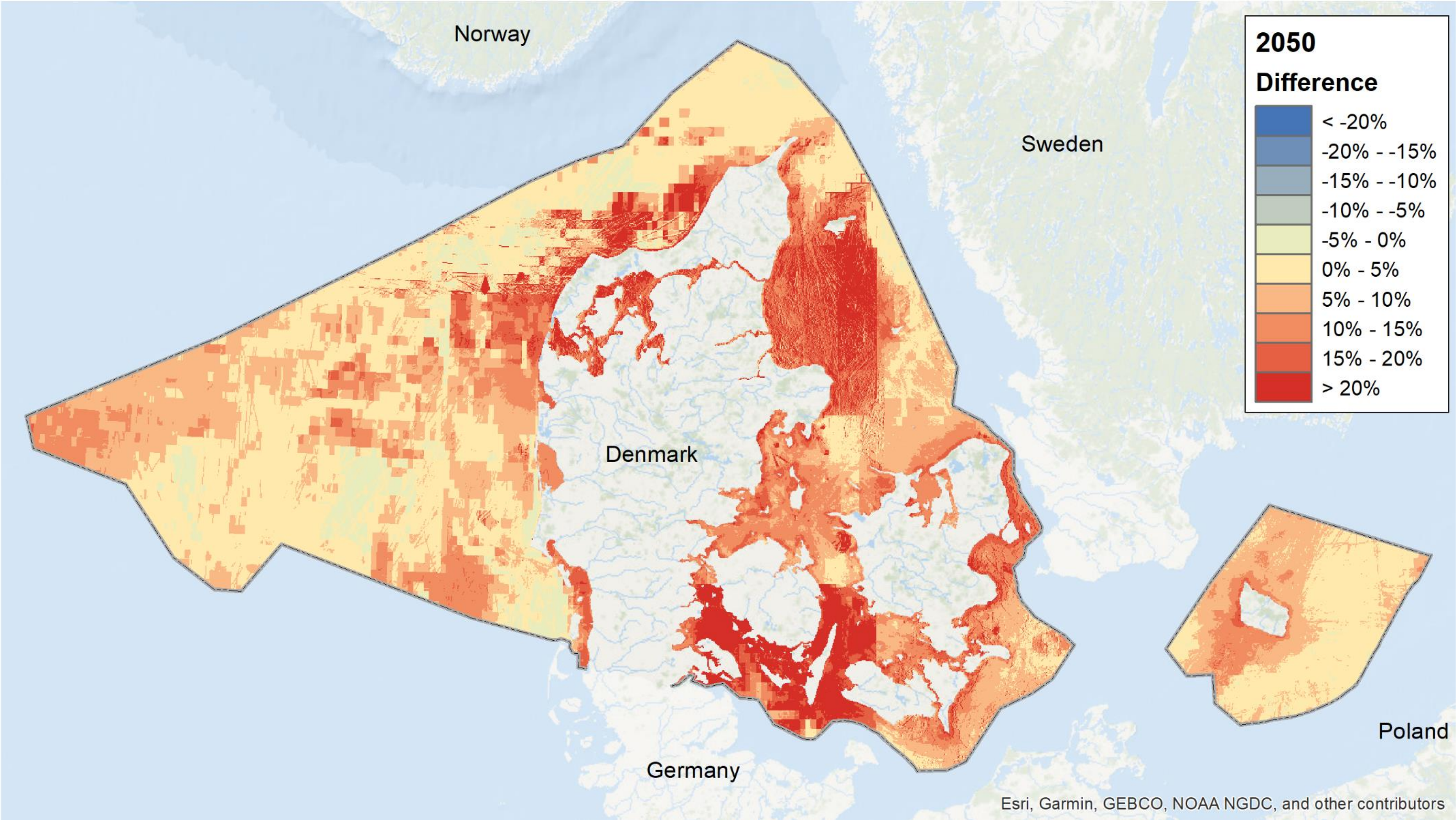

Figure S6.2. Spatial map showing the percentage difference between the baseline current impacts and when the changes in scenario 2050 are applied. See legend for colour coding, where yellow to red colours indicate an increase and green to blue a decrease in human impacts. Image from Andersen et al. (2020b).

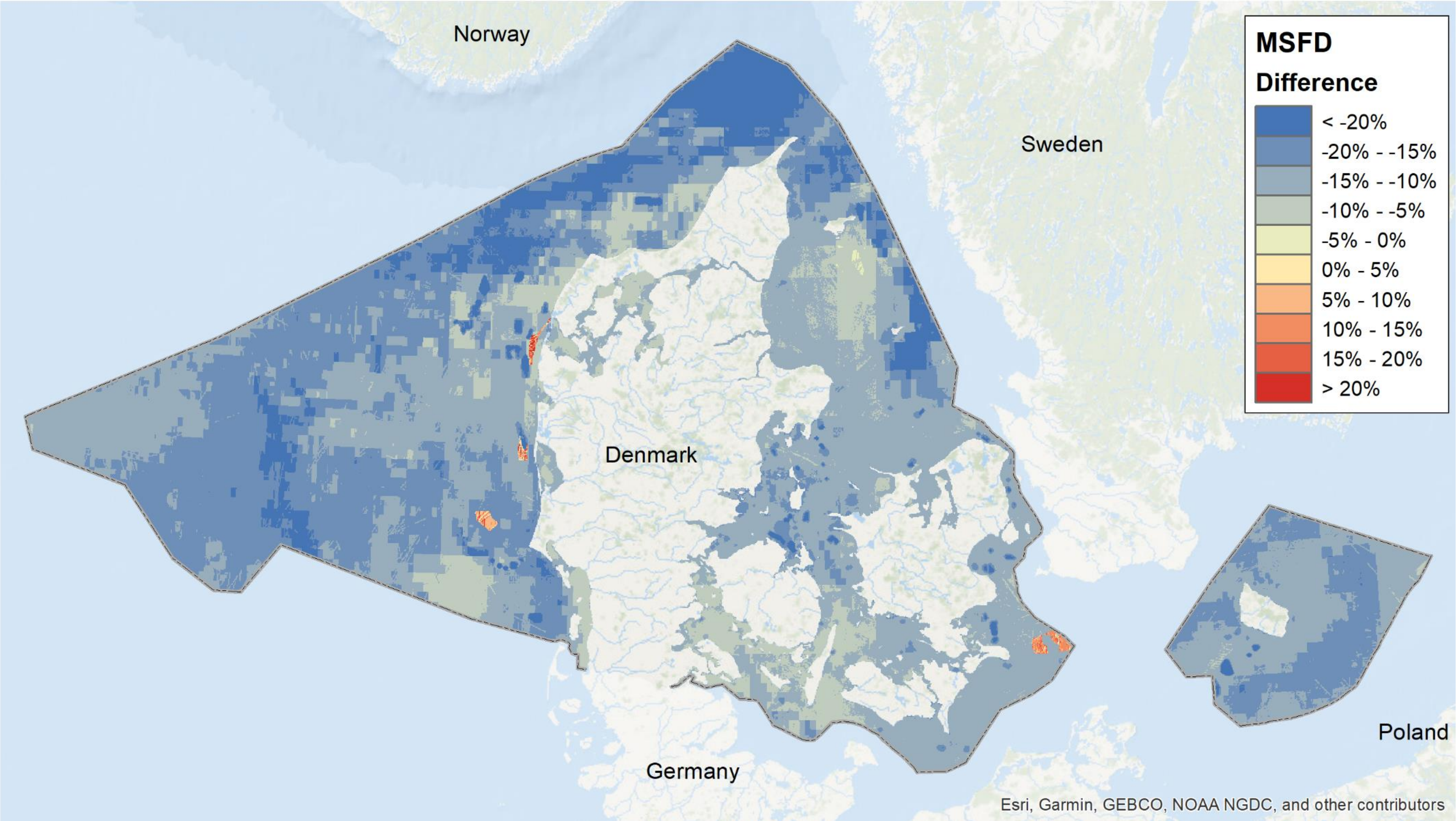

Figure S6.3. Spatial map showing the percentage difference between the baseline current impacts and when the changes in scenario MSFD GEnS are applied. See legend for colour coding, where yellow to red colours indicate an increase and green to blue a decrease in human impacts. Image from Andersen et al. (2020b).

S7: References in Appendix A: Supporting Information

Andersen J.H. (2012): Ecosystem-based management of coastal eutrophication. Connecting science, policy and society. Ph.D. thesis. University of Copenhagen. 56 pp + annexes. http://www2.bio.ku.dk/bibliotek/phd/Jesper%20Andersen.pdf

Andersen, J.H., J. Carstensen, M. Holmer, D. Krause-Jensen & K. Richardson (2019): Editorial. Research and Management of Eutrophication in Coastal Ecosystems. Frontiers in Marine Science 6(768): 1-6. https://www.frontiersin.org/articles/10.3389/fmars.2019.00768/full

Andersen, J.H., Z. Al-Hamdani, E.T. Harvey, E. Kallenbach, C. Murray & A. Stock (2020a): Relative impacts of multiple human stressors in estuaries and coastal waters in the North Sea–Baltic Sea transition zone. Science of The Total Environment 704: 135316. https://www.sciencedirect.com/science/article/pii/S0048969719353082

Andersen, J.H., K.J. Hammer, E.T. Harvey, S.W. Knudsen, C. Murray, J. Carstensen, I.K. Petersen, S. Sveegaard, J. Tougaard, K. Edelvang, J. Egekvist, J. Olsen, M. Vinther, Z. Al-Hamdani, J.B. Jensen, J.O. Leth, B.C. Kaae & A.S. Olafsson (2020b): Supplementary material to ECOMAR: A data-driven framework for ecosystem-based Maritime Spatial Planning in Danish marine waters. NIVA Denmark report, 216 pp. URL: https://niva.brage.unit.no/niva-xmlui/handle/11250/2678968

Andersen, J. H., Bendtsen, J., Hammer, K. J., Harvey, E. T., Knudsen, S. W., Murray, C. J., Carstensen, J., Petersen, I. K., Svegaard, S., Tougaard, J., Edelvang, K., Egekvist, J., Olsen, J., Vinther, M., Al-Hamdani, Z., Jensen, J. B., Leth, J. O., Kaae, B. C., Olafsson, A. S., … Yocum, D. (2020c): ECOMAR: A data-driven framework for ecosystem-based Maritime Spatial Planning in Danish marine waters. Results and conclusions from a development and demonstration project. In 81. Norsk institutt for vannforskning. https://niva.brage.unit.no/niva-xmlui/handle/11250/2725462

Dahllöf, I. & J.H. Andersen (2009): Hazardous and Radioactive Substances in Danish Marine Waters. Status and Temporal Trends. Danish Spatial and Environmental Planning Agency & National Environmental Research Institute. 110 pp. http://www2.dmu.dk/pub/OSPAR_Hazardous_Substances_print.pdf

The Danish Food Administration Agency (2020): https://www.foedevarestyrelsen.dk/Leksikon/Sider/Akvakulturforhold.aspx; https://www.foedevarestyrelsen.dk/Leksikon/Sider/Register-over-danske-akvakulturanl%C3%A6g.aspx

DEA, Danish Energy Agency (2018): https://ens.dk/sites/ens.dk/files/OlieGas/ressourcer_og_prognoser_20180829_rev_en.pdf

EEA (2019): Contaminants in Europe's Seas. European Environment Agency, 61 pp + Online Material. https://www.eea.europa.eu/publications/contaminants-in-europes-seas

EEA (2019) Indicator assessment; Global and European sea-level rise. Prod-ID: IND-193-en. Also known as: CSI 047, CLIM 012. Created 05 Nov 2019 Published 04 Dec 2019 Last modified 04 Dec 2019. Accessed 28 August 2020, from https://www.eea.europa.eu/data-and-maps/indicators/sea-level-rise6/assessment

EEA (2020): Multiple pressures and their combined effects in Europe's seas. ETC/ICM Technical Report 4/2019: European Topic Centre on Inland, Coastal and Marine waters, 164 pp. https://www.eionet.europa.eu/etcs/etc-icm/products/etc-icm-report-4-2019-multiple-pressures-and-their-combined-effects-in-europes-seas
EMODnet (2020): https://emodnet.ec.europa.eu/en/human-activities

HELCOM (2018): State of the Baltic Sea – Second HELCOM holistic assessment 2011-2016. Baltic Sea Environment Proceedings 155. Helsinki Commission. 155 pp. file:///C:/Users/JHA/Downloads/HELCOM_State-of-the-Baltic-Sea_Second-HELCOM-holistic-assessment-2011-2016%20(6).pdf

ICES (2022): ICES Data Centre – link: http://ocean.ices.dk

IPCC (2019): Technical Summary [H.-O. Pörtner, D.C. Roberts, V. Masson-Delmotte, P. Zhai, E. Poloczanska, K. Mintenbeck, M. Tignor, A. Alegría, M. Nicolai, A. Okem, J. Petzold, B. Rama & N.M. Weyer (eds.)]. In: IPCC Special Report on the Ocean and Cryosphere in a Changing Climate, 40-69. https://www.ipcc.ch/site/assets/uploads/sites/3/2019/11/04_SROCC_TS_FINAL.pdf

Kaae, B.C., A.S. Olafsson & H. Draux (2018): Blåt friluftsliv i Danmark. Institut for Geovidenskab og Naturforvaltning, Københavns Universitet. 174 pp. http://ign.ku.dk/formidling/publikationer/rapporter/

Meier, M.E.H. (2015): Projected Change – Marine Physics. In: The BACC II Author Team, Second Assessment of Climate Change for the Baltic Sea Basin. Springer International Publishing AG. Cham, Heidelberg, New York, Dordrecht, London.

Miljø- og Fødevareministeriet (2019): Danmarks Havstrategi II. Første del. God miljøtilstilstand. Basisanalyse. Miljømål. ISBN: 978-87-93593-73-2. 309 pp. https://mfvm.dk/fileadmin/user_upload/MFVM/Natur/Havstrategi/HSII_foerste_del_-_endelig_udgave.pdf

Miljøstyrelsen (2020): Strategi for bæredygtig udvikling af akvakultursektoren i Danmark 2014- 2020. https://mst.dk/erhverv/akvakultur/akvakulturstrategi2014-2020; https://mst.dk/media/147844/strategi_for_baeredygtig_udvikling_af_akvakultursektoren_i_danmark_2014-2020.pdf

NIRAS (2018): Fremskrivning af råstofforbruget 2016-2040 – Region Sjælland. http://rs.viewer.dkplan.niras.dk/media/193996/Fremskrivning-RegionSjaelland.pdf

ODA (2022): Joint database of the Ministry of Environment and Food of Denmark and DCE - Danish Centre for Environment and Energy, Aarhus University, for surface water data – link: https://odaforalle.au.dk

Riemann, B., J. Carstensen, K. Dahl, H. Fossing, J.W. Hansen, H.H. Jakobsen, A.B. Josefson, D. Krause-Jensen, S. Markager, P. Stæhr, K. Timmerman, J. Windolf & J.H. Andersen (2016): Recovery of Danish coastal ecosystems after reductions in nutrient loading: a holistic Ecosystem Approach. Estuaries & Coasts 39(1): 82-97. https://link.springer.com/content/pdf/10.1007/s12237-015-9980-0.pdf

Schrum, C., J. Lowe, H.E.M. Meier, I. Grabemann, J. Holt, M. Mathis et al. (2016): Projected Change - North Sea. In: Quante, M. & F. Colijn (Eds.): North Sea Region Climate Change Assessment. Springer International Publishing, 175–217.

Stæhr P.A., H.H. Jakobsen, J.L.S. Hansen, P. Andersen, M. Storr-Paulsen, J. Christensen, S. Lundsteen, C. Göke, & M.-C. Carausu (2016): Trends in records and contribution of non-indigenous species (NIS) to biotic communities in Danish marine waters. Aarhus University, DCE – Danish Centre for Environment and Energy, 45 pp. Scientific Report from DCE – Danish Centre for Environment and Energy No. 179. 48 pp. https://dce2.au.dk/pub/sr179.pdf

Stæhr, P.A., C. Göke, A.M. Holbach, D. Krause-Jensen, K. Timmermann, S. Upadhyay & S.B. Ørberg (2019): Habitat Model of Eelgrass in Danish Coastal Waters: Development, Validation and Management Perspectives. Frontiers in Marine Science. https://doi.org/10.3389/fmars.2019.00175

DRAFT
